# Characterization of Asymptomatic Bacteriuria *Escherichia coli* Isolates in Search of Alternative Strains for Efficient Bacterial Interference against Uropathogens

**DOI:** 10.3389/fmicb.2018.00214

**Published:** 2018-02-14

**Authors:** Christoph Stork, Beáta Kovács, Barnabás Rózsai, Johannes Putze, Matthias Kiel, Ágnes Dorn, Judit Kovács, Szilvia Melegh, Andreas Leimbach, Tamás Kovács, György Schneider, Monika Kerényi, Levente Emödy, Ulrich Dobrindt

**Affiliations:** ^1^Institute of Hygiene, University of Münster, Münster, Germany; ^2^Department of Medical Microbiology and Immunology, University of Pécs, Pécs, Hungary; ^3^First Department of Internal Medicine, University of Pécs, Pécs, Hungary; ^4^Department of Paediatrics, University of Pécs, Pécs, Hungary; ^5^Enviroinvest Zrt., Pécs, Hungary

**Keywords:** asymptomatic bacteriuria, *Escherichia coli*, bacterial interference, competitiveness, comparative genomics, whole genome draft sequences, fitness, urine

## Abstract

Asymptomatic bacterial colonization of the urinary bladder (asymptomatic bacteriuria, ABU) can prevent bladder colonization by uropathogens and thus symptomatic urinary tract infection (UTI). Deliberate bladder colonization with *Escherichia coli* ABU isolate 83972 has been shown to outcompete uropathogens and prevent symptomatic UTI by bacterial interference. Many ABU isolates evolved from uropathogenic ancestors and, although attenuated, may still be able to express virulence-associated factors. Our aim was to screen for efficient and safe candidate strains that could be used as alternatives to *E. coli* 83972 for preventive and therapeutic bladder colonization. To identify ABU *E. coli* strains with minimal virulence potential but maximal interference efficiency, we compared nine ABU isolates from diabetic patients regarding their virulence- and fitness-associated phenotypes *in vitro*, their virulence in a murine model of sepsis and their genome content. We identified strains in competitive growth experiments, which successfully interfere with colonization of ABU isolate 83972 or uropathogenic *E. coli* strain 536. Six isolates were able to outcompete *E. coli* 83972 and two of them also outcompeted UPEC 536 during growth in urine. Superior competitiveness was not simply a result of better growth abilities in urine, but seems also to involve expression of antagonistic factors. Competitiveness in urine did not correlate with the prevalence of determinants coding for adhesins, iron uptake, toxins, and antagonistic factors. Three ABU strains (isolates 61, 106, and 123) with superior competitiveness relative to ABU model strain 83972 display low *in vivo* virulence in a murine sepsis model, and susceptibility to antibiotics. They belong to different phylogroups and differ in the presence of ExPEC virulence- and fitness-associated genes. Importantly, they all lack marked cytotoxic activity and exhibit a high LD50 value in the sepsis model. These strains represent promising candidates for a more detailed assessment of relevant fitness traits in urine and their suitability for therapeutic bladder colonization.

## Introduction

Acute as well as chronic or recurrent urinary tract infections (UTIs) are a wide spread public health problem and lead to a considerable diminution of quality of life (Jarvis, 1996; Cassini et al., 2016). A total of 50% of all women experience a symptomatic UTI episode at least once in a lifetime, which requires antibiotic treatment (Foxman, 2014). UTI are responsible for up to 11 million medical visits and 100,000 hospital admissions per year in the United States (Russo and Johnson, 2003; Foxman, 2014). A total of 30% of the affected women suffer from chronic recurrent UTI, which often require repeated antibiotic treatment, thus promoting the accumulation of antibiotic resistance in uropathogens. Antibiotic treatment is often still effective, but with recurring symptomatic episodes or long-term prophylaxis often problematic side effects occur, like the selection of antibiotic-resistant variants, destruction of the normal microbiota of the patient, gastrointestinal side effects as well as allergic reactions. Rising rates of antibiotic resistance can lead to increased morbidity and mortality due to inappropriate and inefficient treatment. UTI, one of the most frequently occurring bacterial infections, is a serious global health problem affecting millions of people each year, which is typically caused by a single bacterial species colonizing the urinary tract. A variety of bacteria can cause these infections, which are most often treated with antibiotics. Recurrent episodes are common and infections frequently tend to become chronic and difficult to treat. Bacteria usually enter the urinary bladder through the urethra, causing an initial infection (cystitis). Furthermore, the bacteria can ascend from the lower urinary tract to the upper urinary system through the ureters and infect the kidneys (pyelonephritis). The infection of the kidneys can result in dysfunction or renal failure and may ultimately lead to the dissemination of bacteria into the bloodstream (sepsis). Besides provoking symptoms and eliciting protective host immune responses, *Escherichia coli* can also asymptomatically colonize the human bladder. During asymptomatic bacteriuria (ABU), high bacterial numbers are present in the urinary bladder, without causing symptoms or overt signs referable to UTI. Asymptomatic colonization of the urinary bladder does, with a few exceptions, not require treatment (Cai et al., 2017). Bladder colonization by ABU strains can prevent symptomatic UTI by more virulent bacterial strains (Wullt et al., 1998; Sundén et al., 2010; Köves et al., 2014). The prevalence of ABU varies with age, gender, sexual activity, and anomalies of the genitourinary system. The frequency also depends on individual predispositions and disorders that promote asymptomatic UTI. In general, ABU is uncommon in pediatric patients, while patients with either type 1 or type 2 diabetes mellitus have a higher prevalence of UTI than healthy patients, irrespective of age and gender (Nicolle et al., 2006).

A total of 80% of all uncomplicated outpatient-acquired UTI are caused by *E. coli*. In addition, UTI (catheter-associated) caused by *E. coli* is now among the most frequent nosocomial infections, which are often caused by antibiotic-resistant strains. Globally, the resistance situation in uropathogens is precarious (Flores-Mireles et al., 2015). As an alternative to antibiotic treatment, the deliberate colonization of the urinary bladder with ABU *E. coli* isolate 83972 has been demonstrated and clinical use of isolate 83972 was endorsed in the European Urology Guidelines (Hull et al., 2000; Sundén et al., 2010; Darouiche et al., 2011; Darouiche and Hull, 2012; Rudick et al., 2014; Grabe et al., 2015). Deliberate asymptomatic bladder colonization can be carried out over a long period without side effects (Köves et al., 2014). The high proportion of antibiotic-resistant pathogens in UTI, including chronic-recurring UTI courses, requires a rapid investigation and establishment of alternative treatment strategies for UTI. Though model ABU strain *E. coli* 83972 is a safe and efficient competitor to uropathogenic strains, we feel that it is crucial to extend the search for alternative strains for bacterial interference. It was our goal to identify and characterize further suitable ABU isolates with superior fitness in urine and a reduced pathogenic potential relative to ABU model strain 83972, which could be considered as possible alternatives to *E. coli* 83972 for preventive or therapeutic use in bacterial interference. Our results will stimulate a broader scale search for alternative competitors against a larger collection of uropathogens. We also deem it important to gain comprehensive knowledge of bacterial traits which are required or beneficial for efficient growth and competitiveness in urine. Such traits could be promising targets for antibacterial therapy during UTI.

## Materials and Methods

### Ethics Statement

This study was carried out in accordance with the recommendations of Hospital Ethics Committee with written informed consent from all subjects. All subjects gave written informed consent in accordance with the Declaration of Helsinki. The protocol was approved by the Hospital Ethics Committee. Written informed consent, which is mandatory by Hungarian law for any laboratory or imaging intervention, was obtained from all participants/legal representatives. Accordingly, written consent was given also for urine bacteriology cultures. The urine samples were taken in line with the clinical assessment, and no extra medical intervention was involved.

### Bacterial Strains and Culture Conditions

Diabetic children and adolescents were annually hospitalized for assessment, re-education, and screening for microvascular complications. This opportunity was used to analyze urine bacteriological cultures. After cleaning the genitalia and perineum of patients with three gauze sponges saturated by octenidine dihydrochloride, midstream clean voiding morning urine samples were collected on two consecutive days. The urine sample collection was performed (in patients younger than 12 years of age) or supervised (in patients older than 12) by qualified nurses. Samples were cultured at 37°C overnight on blood agar and eosin methylene blue agar plates, and quantified by the number of colonies. ABU was established by positive *E. coli* cultures (> 10^5^ colonies/ml) in both consecutive samples.

The *E. coli* strains used in this study are listed in **Supplementary Table S1**. ABU *E. coli* strains were isolated in the time period from October 2010 to March 2012 from urine samples of eight female patients with type 1 diabetes mellitus (age range 12–25 years, median 19.5 years) attending yearly controls at the Department of Paediatrics, Division of Endocrinology and Metabolic Diseases, at the University Hospital Pécs (Pécs, Hungary) as well as from one female 52-year-old university co-worker (University Hospital Pécs) suffering from type 2 diabetes. The patients presented with ABU one to five times, respectively, at the yearly samplings (**Supplementary Table S2**) without having clinical symptoms of UTI. None of the patients received antibiotics prior to culturing the ABU strain. ABU *E. coli* isolates were stored at -80°C for further characterization. For analysis, the strains were routinely grown in lysogeny broth (LB), M9 or M63B1 glucose medium (Sambrook et al., 1989) with or without 1.5% Bacto-Agar (Difco Laboratories Inc., Detroit, MI, United States).

### Genome Sequencing

#### Next Generation Sequencing Using the Illumina MiSeq Sequencer

The nine ABU strains were sequenced using Illumina MiSeq technology as described in Stork et al. (2018). Strain ABU65 was also sequenced on a Roche GS Junior sequencer with Titanium chemistry (Roche Hungary Ltd., Budaörs, Hungary) after nebulization and end repair of the genomic DNA (Roche Junior Titanium kit) (Stork et al., 2018). The sequencing raw data have been deposited at the NCBI Sequence Read Archive with the following accession numbers: strain 1 (SRR6224581), strain 9 (SRR6224582), strain 61 (SRR6224583), strain 65 (SRR6224584, SRR6224588, SRR6224589, SRR6224590, SRR6224591), strain 84 (SRR6224579), strain 91 (SRR6224580), strain 106 (SRR6224585), strain 123 (SRR6224586), and strain 148 (SRR6224587).

#### Quality Control and *de Novo* Assembly of Raw Sequence Reads

Raw paired end reads were quality checked using FastQC (v11.5) (Andrews, 2010). Prior to assembly, the raw reads were quality filtered using Sickle (v1.33) (Joshi and Fass, 2011). To reduce the number of false k-mers and improve the assembly process, we used a quality cutoff of 20 and a length limitation of 127 bp. Quality filtered whole genome sequence data of individual isolates were *de novo* assembled with SPAdes (v3.9.0) (Bankevich et al., 2012). Parameter ‘—careful’ and various k-mer lengths (31, 55, 77, 99, 127) were used to minimize the number of mismatches in the final contigs and obtain optimal results. In case of ABU isolate 65, Illumina and GS Junior 454 (Roche) reads were combined for *de novo* genome assembly. The resulting genome assemblies were analyzed using QUAST (v4.3) (Gurevich et al., 2013) and contigs smaller than 1 kb were discarded. Between 43 and 197 contigs were obtained for the individual genomes, accounting for total genome sizes ranging from 4,578,015 to 5,285,335 bp (Stork et al., 2018).

#### Annotation and Determination of Orthologous Genes

The tools Prokka (v1.12) (Seemann, 2014) and Proteinortho (v5.15) (Lechner et al., 2011) were used in order to determine coding sequences (CDS), annotate the genomes and to detect orthologous genes. The two freely available perl scripts ‘cds_extractor’ (v0.7.1) and ‘po2group_stats’ (v.0.1.3) (Leimbach, 2016a) were included in a custom pipeline^1^ to extract amino acid sequences from the CDS features and to categorize orthologs from the Proteinortho output according to genome groups, respectively.

#### Draft Genome Comparison and Typing

The identification of plasmids, serotypes as well as acquired resistance genes was performed with the web-based tools PlasmidFinder (v1.3) (Carattoli et al., 2014), SerotypeFinder (v1.1) (Joensen et al., 2015), and ResFinder (v2.1) (Zankari et al., 2012), respectively. We used a stringent identity threshold of 95% to determine plasmids based on replicon sequences. To examine serotypes and acquired resistance genes a sequence identity of 85 and 90% was used, respectively. The length requirement was set to a minimum of 60% sequence coverage for both, serotyping and identification of resistance genes. For the gene-by-gene comparison, we performed a pan-genome analysis using Roary v3.7.0 (Page et al., 2015) to determine the sizes of the core and pan-genomes of all re-isolates and 20 additional strains that represent the major phylogenetic lineages of the *E*. *coli* collection of reference strains (ECOR). For core genome multilocus sequence typing (cgMLST) based on the Roary core genome alignment, we used RAxML (Stamatakis, 2014) with the corresponding consensus files generated for each ABU strain. The core genome comprised 1,579 genes conserved in > 99% of all strains included into the comparison^2^.

#### Identification of Virulence-Associated Factors Encoded by the Different ABU Isolates

For the determination of virulence factors (VFs), we extended the *E. coli* VF collection (v0.1) (Leimbach, 2016b). In total, the final collection comprises 12 distinct VF groups containing 1,154 deduced protein sequences of virulence-associated genes. An automated search for protein homologs in annotated bacterial genomes and the identification of present or absent VFs were processed with the ‘prot_finder’ pipeline of the ‘bac-genomics-scripts’ collection using BLASTP+ (Leimbach, 2016a). The heatmap of the corresponding binary presence/absence matrix was generated with the statistical computing and graphics software environment R (v3.2.2) (R Core Team, 2017). The virulence binary matrix was used to examine the relationship of present/absent virulence- and fitness-associated genes with the phylogroup and sequence type via multivariate analyses. A principle coordinates analysis (PCoA) was plotted based on a Bray-Curtis similarity matrix of the BLASTP hits with PAST (v3.18) and a transformation exponent of *c* = 2 (Legendre and Legendre, 1998; Hammer et al., 2001). PCoA allows to maximally correlate the distances in the ordination diagram with the linear distance measures in the distance matrix. Here, the PCoA was used to examine the grouping of strains according to the virulence- and fitness gene matrix and their competitiveness in urine (Quinn and Keough, 2002; Ramette, 2007).

### Phenotypic Analysis

#### Hemolytic Capacity

The strains were streaked out on Columbia agar plates containing 5% sheep blood (Oxoid, Wesel, Germany), and the presence of hemolytic zones was checked after 24 h incubation at 37°C.

#### Cytotoxicity on T24 Uroepithelial Cells

The T24 uroepithelial cells were seeded in 96-well tissue culture plates, grown overnight, and washed with antibiotic-free tissue culture fluid. The 10^8^ colony forming units (CFU) of washed bacteria grown over night in LB were added to the wells, and incubated for 3 h. After three washing steps in phosphate-buffered saline (PBS), 2% formaldehyde was added for 1 min, and the cells were washed with PBS once again. Staining of T24 cells was performed with 0.13% crystal violet, and the cells were lysed with 1% SDS solution. Cell-bound crystal violet was quantified at 595 nm. Each individual strain was assayed in triplicate. Error bars indicate SDs. Uropathogenic *E. coli* (UPEC) wild type strain 536 was used as a positive control. The α-hemolysin-negative mutant *E. coli* 536-21 served as a negative control (Hacker et al., 1986).

#### Adherence to T24 Uroepithelial Cells

The 3 × 10^5^ T24 human bladder epithelial cells were seeded into each well of a 24-well plate, and cells were allowed to adhere overnight. The 10^7^ CFU of washed bacteria (grown over night in LB) were added, and the plates were incubated at 37°C in 5% CO_2_ for 1 h to allow adhesion of the bacteria. The T24 cell monolayers were washed three times with PBS to remove non-adherent bacteria. For quantification of adherent bacteria, the T24 cells were lysed by the addition of 200 μl of 1% Triton X-100 in PBS for 10 min. Bacterial viable counts were determined by serial dilution of samples followed by plating onto LB agar plates. The number of adherent bacteria was formulated as percentage of the total bacterial number added at the beginning of the incubation period (10^7^ CFU). Averages ± SDs were calculated from four independent experiments. UPEC strain 119 (Kuch et al., 1982) served as a positive control and *E. coli* strain 536-21 as a negative control.

#### Adherence to Extracellular Matrix Proteins

The extracellular matrix proteins collagen type I, collagen type IV, laminin, and fibronectin (Sigma–Aldrich, Budapest, Hungary) were diluted to 20 μg/ml concentration. The 96-well microtiter plates were coated with 100 μl of the protein solutions, and incubated for 16 h at 4°C. Every well was washed three times with 200 μl PBS-Tween (0.05% Tween-20, pH 7.0). Unspecific binding was blocked with 100 μl of 2% bovine serum albumin (BSA) for 2 h at 25°C. The ABU and control *E. coli* isolates were cultured in 5 ml of LB for 16 h at 37°C and the optical density was adjusted to 0.58 at 555 nm (10^9^ CFU/ml). After the elimination of BSA from the protein-coated wells by three washing steps with 200 μl PBS-Tween solution, each well was filled with 100 μl of bacterial suspension. After an incubation period of 90 min, the wells were washed with 200 μl PBS-Tween solution to remove non-adherent bacteria. Adherent bacteria were resuspended in 100 μl 0.1% Triton X-100 solution and bacterial counts were assessed on LB agar plates. Each individual strain was assayed in triplicate. Error bars indicate SDs. *E. coli* TB1 (pC4003) and TB1 (pUC19) strains served as positive and negative controls, respectively (Kienle et al., 1992).

#### Siderophore Production

The production of secreted siderophores was visualized on chrome azurol S (CAS) indicator agar plates (Schwyn and Neilands, 1987) supplemented with 1 μg/ml nicotinamide upon overnight incubation at 37°C. Expression of the siderophores enterobactin and aerobactin was determined by cross-feeding assays. Strains were grown in liquid LB overnight at 37°C, with 200 rpm, washed in PBS, and resuspended in PBS to ∼10^6^ cells/ml. LB plates containing 320 mM 2,2′-dipyridyl were seeded with the indicator strains, and when dried 1 μl suspension of the test strains was dropped. Cross-feeding was observed after incubation at 37°C, for 24 and 48 h. Indicator strains are as follows: enterobactin receptor-positive *E. coli* strain H1939 and aerobactin receptor-positive *E. coli* strain H1887. Control strains are as follows: *E. coli* K311 aerobactin (+), enterobactin (+), *Klebsiella pneumoniae* 390 aerobactin (-), enterobactin (+), *K. pneumoniae* W122 aerobactin (-), and enterobactin (-).

#### Bacteriocin Production

The production of small secreted antagonistic factors such as bacteriocins was tested in a modified soft agar overlay assay (Fredericq, 1957). The different *E. coli* isolates were spotted onto M9 soft agar (0.75% agar), which was seeded evenly with an *E. coli* indicator strain (1 ml overnight culture per 100 ml soft agar). After drying, the test ABU strains were spotted on the plates in proper distances. By next day, growth of the seeded indicator strain forms a continuous lawn covering the surface of the plate. Growth of the spotted ABU *E. coli* isolates and secretion of antagonistic factors into the agar interferes with growth of the indicator strain. Consequently, a clear halo indicating growth inhibition of the indicator strain will appear in the overlay agar around the producing colony. *E. coli* strain DH5α, ABU isolate 83972, and UPEC strain 536 have been used as indicator strains.

#### Biofilm Formation

Bacterial strains were cultivated overnight in LB cultures (37°C, 200 rpm) before fresh medium in 96-well tissue culture plates was inoculated with ∼5 × 10^7^ bacteria/well and incubated for 18 h at 37°C. Similarly, overnight cultures grown statically in pooled human urine (37°C) were also used to inoculate round-bottom 96-well PVC plates with 5 × 10^7^ bacteria/well and then incubated for 48 h at 37°C. After the incubation, culture medium and non-bound bacteria were discarded by washing with PBS, and attached cells were fixed with 2% formaldehyde. After staining with 0.13% crystal violet solution for 30 min, excess stain was removed by three wash steps with PBS. The biofilm-bound crystal violet was solubilized by the addition of 200 μl 80% ethanol, 20% acetone, and quantified photometrically at a wavelength of 595 nm. *E. coli* strains 83972, 536 as well as K-12 strain MG1655 were used as references. Each value is an average of three biological replicates measured in two technical replicates. Error bars indicate SDs. Expression of the ‘red dry and rough’ (rdar) multicellular behavior at 30 and 37°C was visualized by cultivation for 48 h on LB without salt agar supplemented with 40 μg/ml Congo Red (Sigma–Aldrich, Deisenhofen, Germany) and 20 μg/ml Coomassie Brilliant Blue G-250 (Sigma–Aldrich, Deisenhofen, Germany) (Römling et al., 1998). Duplicates were used for each strain, and assays were repeated three times.

#### Serum Resistance

Bacteria grown in LB were washed in saline and diluted to 10^6^ CFU/ml. A total of 100 μl aliquots of bacterial suspensions were mixed with an equal volume of human serum, and incubated at 37°C for 3 h in microtiter plates. Samples were taken at 0, 1, and 3 h. Viable cell counts were determined by plating aliquots onto LB plates and overnight incubation at 37°C. The assays were performed both with normal and with heat-inactivated (56°C for 30 min) serum. Duplicates were used for each strain, and assays were repeated three times. Error bars indicate SDs. *E. coli* strain 536 was used as a positive control. *E. coli* strain 536-21 served as a negative control.

#### Intravenous Mouse Virulence Assay

Animal experiments were conducted in accordance with the 2010/63 EU directive and the Hungarian governmental decree No. 328/2010 (paragraphs 1–3) with a license number of BA02/2000-21/2011 for the Department of Medical Microbiology and Immunology, University of Pécs. The 8-week-old specific pathogen-free female BALB/c mice were purchased from Charles River (Hungary). Overnight LB cultures of bacteria were washed twice in PBS, and normalized to 10^9^ cells/ml by optical density in PBS. Graduated doses of fivefold dilutions were given to mice into the tale vein. Observation was continued for 14 days, and death was recorded daily.

#### Measurement of Monoculture Growth in Pooled Human Urine

Monocultures of the *E. coli* included into this study were cultivated over night at 37°C and 180 rpm in 2 ml pooled human urine in an Infors HT Multitron Standard incubator (Infors, Einsbach, Germany). These overnight cultures were used to inoculate fresh pooled human urine to an optical density of 0.01 at 595 nm and 150 μl of these cultures were placed in microtiter plates with transparent bottom (Greiner Bio-One, Frickenhausen, Germany), covered with a transparent lid. Afterward the plate was incubated at 37°C in a TECAN Infinite F200 instrument (TECAN, Männedorf, Switzerland) for 8 h with orbital shaking with an amplitude of 2 mm. Optical density at 595 nm was recorded automatically every 20 min. The optical density signal was corrected for blank (sterile pooled humane urine). Each value is an average of three biological replicates measured in two technical replicates. Error bars indicate SDs.

#### *In Vitro* Growth Competition Assay

To compare the fitness of the different ABU isolates in LB or pooled human urine, we tested the *in vitro* competitiveness of the individual ABU isolates against archetypal ABU or uropathogenic strains *E. coli* 83972 and 536, respectively. For this purpose 30 ml LB or pooled human urine were inoculated in a 1:1 ratio (OD_600_ = 0.01) with an overnight culture of one of the ABU isolates and either ABU strain 83972 or UPEC strain 536 grown in the same respective medium. These cultures were grown statically at 37°C, and samples were taken at different time points (0, 1, 2, 3, 5, 7, and 24 h). Suitable dilutions were plated on LB agar supplemented with chloramphenicol (20 μg/ml), streptomycin (100 μg/ml), or ampicillin (100 μg/ml) as well as on pure LB agar plates to distinguish between the two competing strains in each culture. Based on the CFU counts in the presence or absence of the antibiotic, the fraction of the chloramphenicol-resistant model strain 83972::*cat*, the streptomycin-resistant UPEC strain 536, or the ampicillin-resistant ABU isolates 65 and 148 in the culture was determined for the different time points. Each value is an average of three biological replicates measured in two technical replicates. Error bars indicate SDs. Uninoculated pooled human urine was also plated to exclude contamination, and yielded no bacterial growth.

## Results

### Phenotypic Characterization of Selected ABU *E. coli* Isolates

**FIGURE 1:**
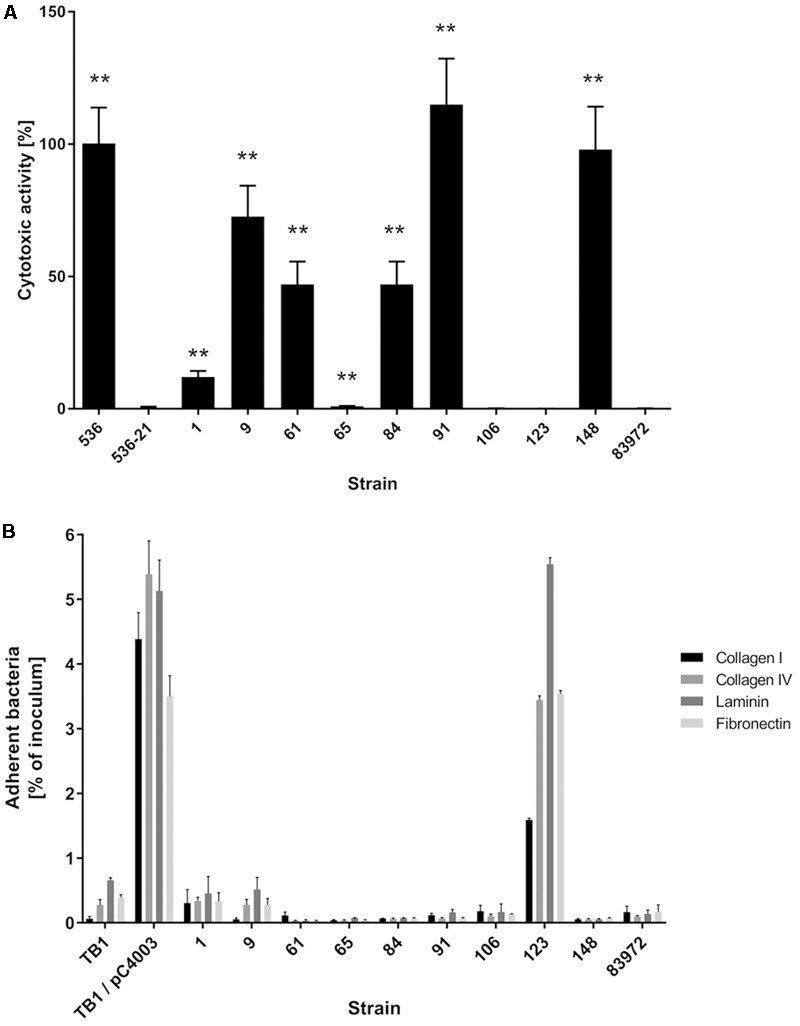
Virulence traits of nine ABU isolates compared with ABU model isolate 83972 or UPEC strain 536. **(A)** Cytotoxicity of the strains to T24 uroepithelial cells as determined by staining of surviving cells with crystal violet. Hemolytic UPEC strain 536 served as positive control, and its non-hemolytic mutant 536-21 served as negative control. Asterisks indicate strains with significantly different cytotoxicity compared to reference strain 83972 (unpaired two-tailed *t*-test; ^∗^*P* < 0.05; ^∗∗^*P* < 0.005; ^∗∗∗^*P* < 0.0005; ^∗∗∗∗^*P* < 0.0001). The data shown in the graph are mean values, the error bars indicate SD. **(B)** Matrix protein binding of ABU isolates to different extracellular matrix proteins quantified as percentage of adherent bacteria relative to the total bacterial number used as inoculum. *E. coli* K-12 strain TB1 served as a negative control, and its plasminogen-binding protein Pla expressing variant TB1/pC4003 served as a positive control. Only the positive control *E. coli* TB1/pC4003 and ABU strain 123 exhibited a significantly higher binding capacity to the four ECM proteins tested compared to ABU model strain 83972 (unpaired two-tailed *t*-test, *P* < 0.0001). The data shown in the graph are mean values, the error bars indicate SD.

**FIGURE 2:**
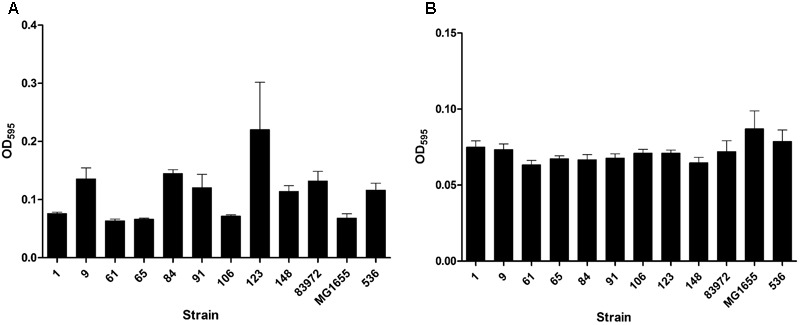
Biofilm formation of nine ABU isolates. The ability to form biofilms in LB **(A)** or in pooled human urine **(B)** at 37°C was compared to that of model strains 83972 (ABU), MG1655 (non-pathogenic), and 536 (UPEC). Biofilms were grown in microtiter plates and quantified by crystal violet staining. Biofilm formation of the individual strains was not significantly increased or decreased compared to reference strain 83972 (unpaired two-tailed *t*-test). The data shown in the graph are mean values, the error bars indicate SD.

Our aim was to search for ABU *E. coli* isolates with a high competitiveness in urine, but a low prevalence of virulence-associated genes and low virulence potential. Accordingly, we tested the collected ABU isolates for the expression of typical UPEC virulence-associated traits. The results of this phenotypic comparison are summarized in **Table 1**. Four ABU isolates were hemolytic, and a halo resulting from complete lysis of erythrocytes was observed on Columbia blood agar plates for ABU strains 9, 84, 91, and 148 (**Supplementary Figure S1A**). To further extend the screening for cytotoxic factors released by the *E. coli* isolates, we tested their toxicity to T24 uroepithelial cells. Only the ABU isolates 9, 91, and 148 presented with marked cytotoxicity while the other tested strains, including the prototypic ABU strain 83972, elicited no harm to T24 cells (**Figure 1A**). The ABU isolates 9, 61, 91, and 106 exhibited an adhesive capacity to T24 cells comparable to that of UPEC strain 119 (data not shown). We also tested for bacterial adherence to extracellular matrix proteins. While ABU strain 123 bound to all four investigated matrix proteins (collagen type I, collagen type IV, laminin, and fibronectin), none of the remaining strains, including ABU model isolate 83972, showed affinity to any of the tested compounds (**Figure 1B**). Global siderophore expression has been analyzed on CAS agar plates (**Supplementary Figure S1B**). The extent of siderophore secretion differed markedly between the isolates. The strongest siderophore secretion was observed for ABU isolates 84, 106, and 148, whereas strains 1, 61, 65, and 123 displayed the lowest secretion of siderophores on CAS agar plates (**Supplementary Figure S1B**). Also the three UPEC model strains 536, CFT073 and UTI89 as well as ABU strain 83972 differed markedly in their ability to secrete siderophores. Enterobactin expression was confirmed in ABU strains 1, 84, and 148, while aerobactin was synthesized by the *E. coli* isolates 9, 84, 106, and 148 (data not shown). Bacteriocin secretion was detected for all ABU isolates except strains 1, 61, and 123 (**Supplementary Figure S1C**). The remaining ABU strains inhibited growth of *E. coli* DH5α. The strongest bacteriocin producers, ABU *E. coli* 9 and 106, also inhibited the growth of ABU strain 83972 and UPEC 536 (**Supplementary Figure S1C**). Biofilm formation in LB of the ABU isolates 9, 84, 91, 123, and 148 was higher or comparable to that observed with *E. coli* 83972 or UPEC strain 536 (**Figure 2A**). Biofilm formation in pooled human urine was generally very weak and did not markedly differ between the individual isolates (**Figure 2B**). The rdar multicellular behavior, i.e., the simultaneous expression of curli fimbria and cellulose, was observed for ABU isolates 1, 91, and 106 at 30 and 37°C (**Supplementary Figure S1D**).

**Table 1:** Phenotypic characterization of ABU *E. coli* isolates.

ABU strain	MSHA	MRHA	Adhesion to T24 cells	Matrix protein binding	Hemo-lysis	Cyto-toxicity	Aero-bactin	Entero-bactin	Colicin (*E. coli* DH5α)	Colicin (*E. coli* 536)	Colicin (*E. coli* 83972)	rdar morpho-type (37°C)	Biofilm formation (LB, 37°C)	Serum resistance	LD50 (murine sepsis model)	Competi-tiveness (UPEC 536)^a^	Competi-tiveness (*E. coli* 83972)^a^
1	-	-	-	-	-	-	-	+	-	-	-	+	-	+	> 5 × 10^8^	-	-
9	+	-	+	-	+	+	+	-	+	+	+	-	+	+	10^7^	+	+
61	-	-	+	-	-	-	-	-	-	-	-	-	-	+	≤ 5 × 10^8^	-	+
65	-	-	-	-	-	-	-	-	+	-	-	-	-	-	> 5 × 10^8^	-	+
84	-	-	-	-	+	+	+	+	+	-	-	-	+	-	10^7^	-	-
91	-	-	+	-	+	+	-	-	+	-	-	+	+	+	≤ 5 × 10^7^	-	+
106	-	-	+	-	-	-	+	-	+	+	+	+	-	+	≤ 5x10^8^	+	+
123	-	-	nt	+	-	-	-	-	-	-	-	-	+	-	> 5x10^8^	-	+
148	-	-	nt	-	+	+	+	+	+	-	-	-	+	+	10^7^	-	-
83972	-	-	-	-	-	-	+	+	+	-	-	-	+	-	> 5 × 10^8^	-	nt

*MSHA, mannose-sensitive hemagglutination (type 1 fimbria expression); MRHA, mannose-resistant hemagglutination (P-fimbria expression); nt, not tested. ^*a*^+, increased competitiveness compared to the reference strain (*P* < 0.0001).*

Only ABU strains 1, 106, and 148 resisted to the killing activity of normal human serum for 3 h (**Table 1**). In the murine sepsis model, the *E. coli* ABU strains 1, 65, and 123 as well as model ABU strain 83972 killed no mice even when as many as 5 × 10^8^ bacterial cells were introduced intravenously. Among the other strains, some exhibited a high LD50 value of 5 × 10^8^ bacteria (*E. coli* ABU strains 61 and 106) or the 50% lethal dose was in the range of 10^7^ bacteria (ABU strains 9, 84, 91, and 148) (**Table 1**).

**FIGURE 3:**
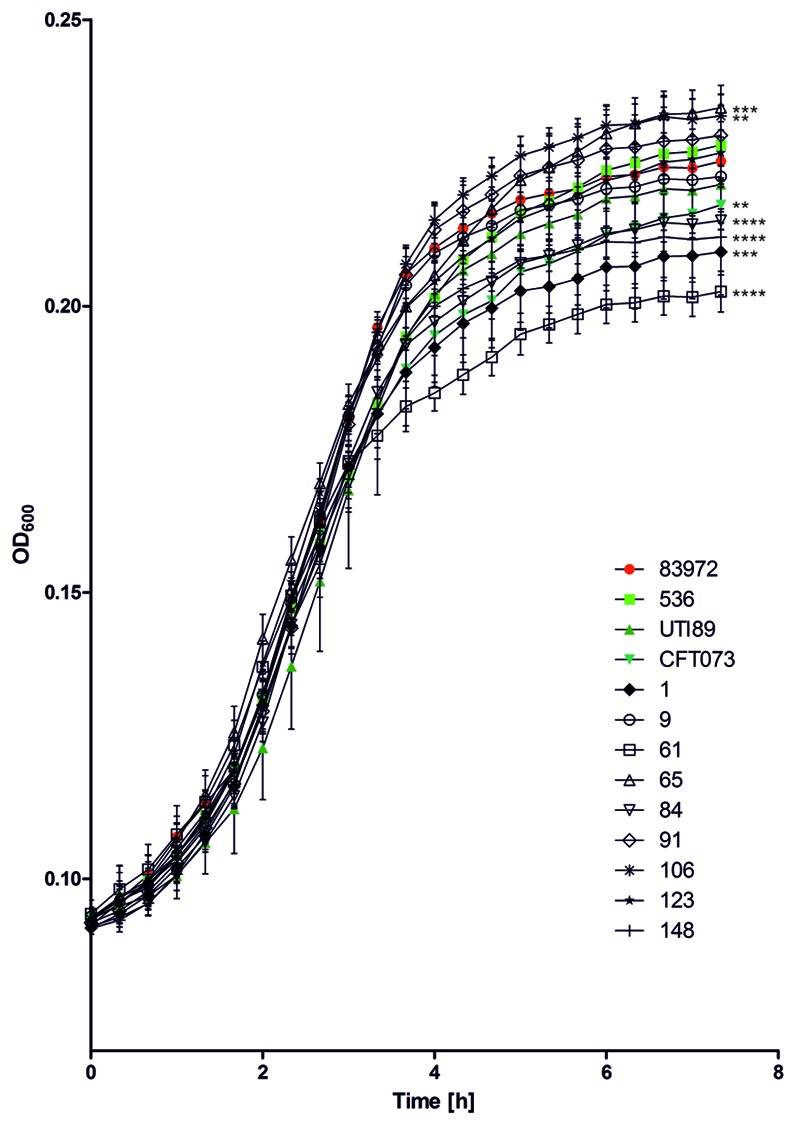
Growth of ABU isolates and selected UPEC strains in pooled human urine. The growth characteristics of the nine ABU isolates from diabetic patients were compared to those of ABU model isolate 83972 and those of archetypal UPEC isolates 536, CFT073, and UTI89. Asterisks indicate strains with significantly different final OD_600nm_ compared to reference strain 83972 (unpaired two-tailed *t*-test; ^∗^*P* < 0.05; ^∗∗^*P* < 0.005; ^∗∗∗^*P* < 0.0005; ^∗∗∗∗^*P* < 0.0001). The data shown in the graph are mean values, the error bars indicate SD.

Growth of the ABU isolates in pooled human urine at 37°C was compared with that of ABU strain 83972 and three UPEC model strains 536, CFT073, and UTI89 (**Figure 3**). The growth rates of all tested strains were very similar in the exponential growth phase. The individual strains differed, however, in the time point when they entered stationary phase as well as in their final optical density. ABU isolates 65 and 106 reached significantly higher optical densities than model ABU strain 83972 (paired two-tailed Mann–Whitney *U*-test, *P* = 0.0022 for ABU 65; *P* = 0.0103 for ABU 106), whereas other UPEC strains or ABU isolates grew to significantly lower cell densities relative to *E. coli* strain 83972 (paired two-tailed Mann–Whitney *U*-test, *P* = 0.0022 for ABU 1, ABU 61, ABU 84; *P* = 0.0043 for CFT073; *P* = 0.005 for ABU 148) (**Figure 3**).

**FIGURE 4:**
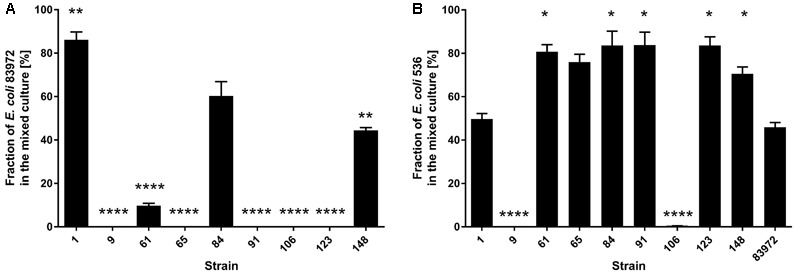
Results of competitive growth experiments between nine ABU isolates and model *E. coli* strains 83972 or 536. The results of competitive growth experiments (mixed cultures, 1:1) between the ABU isolates and either ABU model strain 83972 **(A)** or UPEC strain 536 **(B)** after 24 h of growth in pooled human urine are indicated. Asterisks indicate strains with significantly different competitive fitness compared to reference strains 83972 or 536 (paired two-tailed *t*-test; ^∗^*P* < 0.05; ^∗∗^*P* < 0.005; ^∗∗∗^*P* < 0.0005; ^∗∗∗∗^*P* < 0.0001). The data shown in the graph are mean values, the error bars indicate SD.

Additionally, we compared the competitiveness of the ABU isolates relative to that of ABU model strain 83972 or UPEC strain 536 in pooled human urine. After 24 h of growth, ABU strains 9, 61, 65, 91, 106, and 123 outcompeted *E. coli* 83972 (**Figure 4A**). The strains 9 and 106 were the only ones that also outcompeted UPEC strain 536 under these conditions (**Figure 4B**). *E. coli* 83972 was not able to replace UPEC strain 536 (**Figure 4B**). Investigating the fraction of *E. coli* ABU isolate 83972 in the different mixed cultures over time (**Supplementary Figure S2**), it was obvious that ABU strain 9 efficiently outcompeted *E. coli* 83972 already after 2 h of growth. ABU strains 65, 91, 106, and 123 completely displaced the model ABU strain from mixed cultures after 3 h of growth. ABU strain 61 was the only one that significantly reduced the fraction of *E. coli* 83972 from the mixed urine culture after 24 h (paired two-tailed Mann–Whitney *U*-test, *P* = 0.0022), but could not completely displace the ABU model isolate (**Supplementary Figure S2**).

Taken together, we provide evidence that the ABU isolates analyzed in this study represent a phenotypically diverse group of strains with differential capacity to express extraintestinal pathogenic *E. coli* (ExPEC) virulence- and fitness-associated traits. Several of these isolates were able to outcompete ABU model strain 83972 and/or UPEC model strain 536 in pooled human urine. The superior competitiveness of these strains was not strictly dependent on efficient growth in urine or the expression of siderophores or antagonistic factors. Rather, the competitive advantage in urine results from an individual combination of virulence- and fitness-related traits expressed by these strains.

### Genomic Characterization of Selected ABU *E. coli* Isolates

**FIGURE 5:**
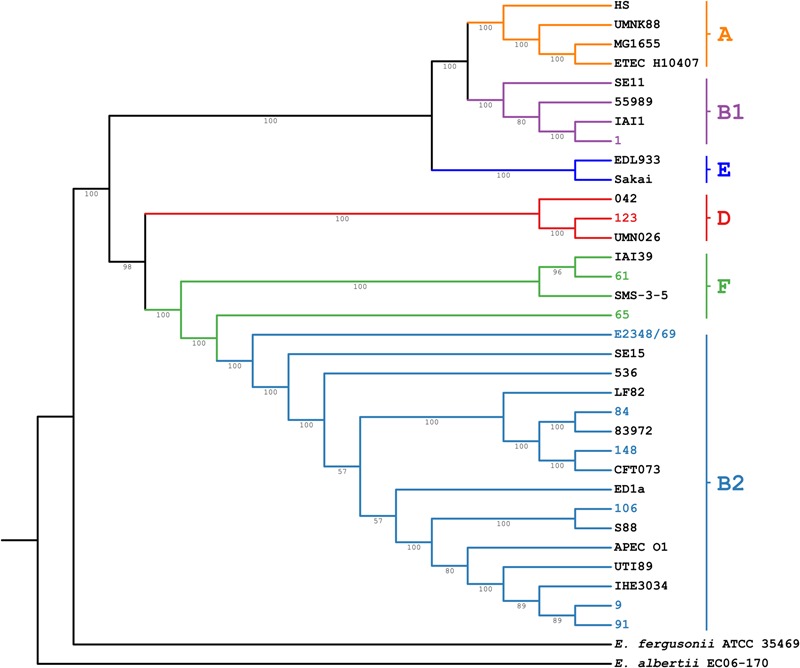
Core genome MLST analysis of the ABU isolates. The neighbor joining tree is based on the analysis of 1,579 open reading frames conserved in the ABU isolates, representative *E. coli* reference strains of the different major phylogroups, and *E. fergusonii* ATCC 35469 and *E. albertii* EC06-170. The ABU isolates analyzed in this study are indicated by different colors.

The genome sequence analysis of the ABU isolates indicated that five out of nine ABU isolates (strains 9, 84, 91, and 106) belonged to phylogroup B2. Two strains were allocated to phylogroup F (ABU strains 61 and 65), whereas ABU strain 123 and 1 belonged to phylogroups D and B1, respectively (**Figure 5** and **Table 2**). The ABU isolates of phylogroup B2 represented two major clonal lineages, i.e., ST73 and ST95. According to the cgMLST, the three ST95 ABU strains 9, 91, and 106 clustered together with typical ExPEC isolates of ST95, e.g., strains IHE3034, UTI89, S88, and APEC_O1. Also the two ST73 ABU isolates 84 and 148 were grouped together with model strains CFT073 and 83972, which belong to the same sequence type (**Figure 5**). The remaining four ABU isolates represent different STs. Besides ABU strain 1, all analyzed ABU isolates harbor at least one plasmid (**Table 2**). Except for ABU strains 61 and 106, the predicted plasmid replicon types found in the individual strains differ markedly in their composition. IncF and colicin plasmids were most prevalent in the isolates.

**Table 2:** Genome sequence-based characterization of the ABU isolates and selected model *E. coli* isolates.

ABU strain	Phylogroup	Serotype	Sequence type	Plasmid replicon sequences	Typical UPEC marker genes	Antibiotic resistance determinants
1	B1	O153/O178:H19	ST 205	–	*fim, ent*	–
9	B2	O18ac:H7	ST 95	IncFIB, IncFIC	*fim, sfa, hly, cdt, clb, cnf, ybt, iuc, iro, ent*	–
61	F	O1:H7	ST 59	IncFIB, IncFII, Col156	*fim, ybt, iuc, ent, sit*	–
65	F	O33:H4	ST 117	IncFIB, IncFIC, IncI1, ColpVC	*fim, ybt, iro, ent, sit*	*strAB, bla*TEM-1B
84	B2	O25:H1	ST 73	IncFII	*sfa*/*foc, pap, hly, clb, cnf, ybt, iuc, iro, ent, sit*	–
91	B2	O18ac:H7	ST 95	IncFIA, IncFIB, IncX1	*fim, sfa, hly, cdt, clb, cnf, ybt, iuc, iro, ent*	–
106	B2	O50/O2:H4	ST 95	IncFIB, IncFII, Col156	*fim, ybt, iuc, iro, ent, sit*	–
123	D	O17/O77:H18	ST 69	Col156	*fim, ent, sit*	–
148	B2	O6:H1	ST 73	Col	*fim, foc, hly, clb, cnf, ybt, iuc, iro, ent, sit*	*strAB, bla*TEM-1B, *sul*2
83972	B2	O25:K5:H1	ST 73	–	*sfa*/*foc, pap, hly, clb, cnf, ybt, iuc, iro, ent, sit*	–
CFT073	B2	O6:K2:H1	ST 73	–	*fim, sfa*/*foc, pap, hly, clb, ybt, iuc, iro, ent, sit*	–
536	B2	O6:K15:H31	ST 127	–	*fim, sfa*/*foc, pap, hly, clb, ybt, iro, ent, sit*	–
UTI89	B2	O18:K1:H7	ST 95	IncFIB, IncFII, Col156	*fim, sfa*/*foc, pap, hly, clb, cnf, ybt, iro, ent, sit*	–

*The following UPEC virulence determinants were considered to be present in the isolates if > 70% of the corresponding genes of the individual determinants have been detected in the genome sequence: *fim*, type 1 fimbrial determinant; *sfa*, S fimbrial determinant; *foc*, F1C fimbrial determinant; *pap*, P-fimbrial determinant; *hly*, α-hemolysin determinant; *cdt*, cytolethal distending toxin determinant; *clb*, colibactin determinant; *cnf*, cytotoxic necrotizing factor determinant; *ybt*, yersiniabactin determinant; *iuc*, aerobactin determinant; *iro*, salmochelin determinant; *ent*, enterobactin determinant; *sit, Salmonella* iron transport system determinant.*

Antibiotic resistance genes were only predicted for ABU isolates 65 and 148. Both strains harbor the *strAB* and *blaTEM-1B* genes, which are associated with resistance against aminoglycosides and β-lactam antibiotics, respectively. These two strains were also phenotypically confirmed to be ampicillin and streptomycin resistant. In addition, strain ABU 148 carries the *sul2* gene, which confers resistance against sulfonamides.

**FIGURE 6:**
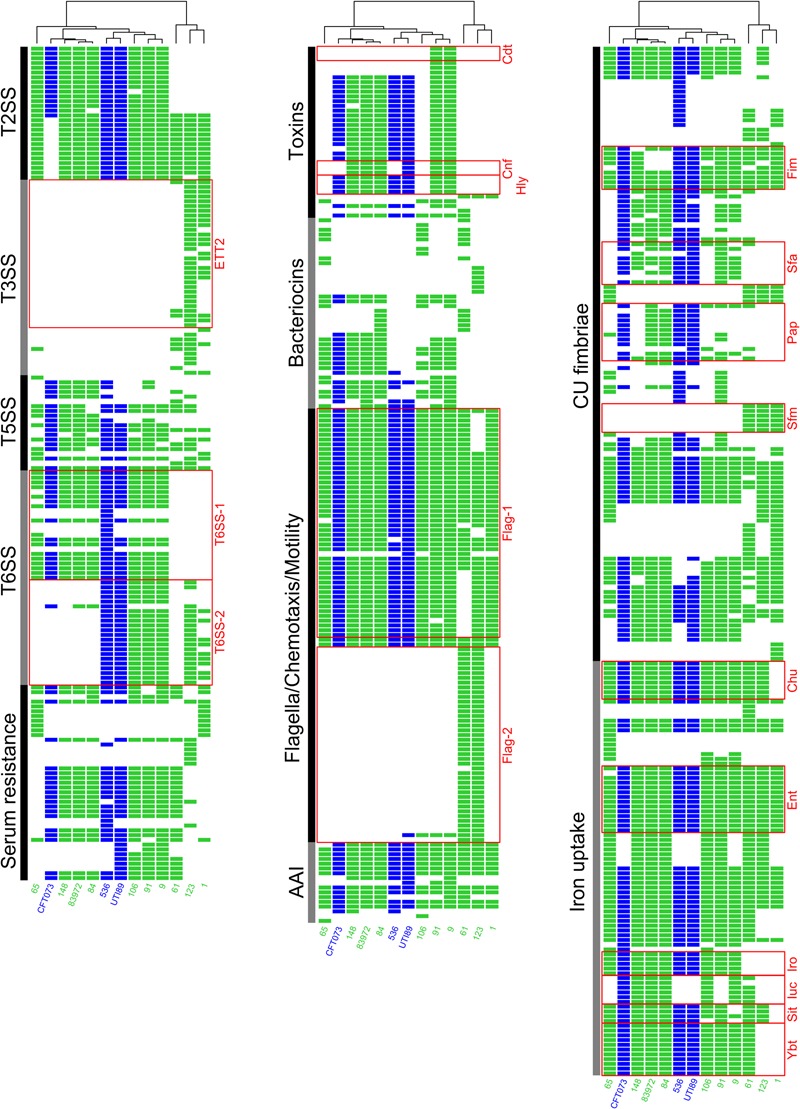
Heatmap indicating the presence or absence of ExPEC virulence- and fitness-associated factors. Each row of the binary matrix indicates the presence or absence of a virulence-associated gene (a BLASTP+ hit). Virulence factor classes are indicated at the side in black and gray. Strain names and individual columns are color-coded for ABU isolates (green) or UPEC strains (blue). Well-known ExPEC virulence determinants are indicated by red boxes. The clustering dendrogram attached to the heatmaps is based on the whole binary dataset of a best scoring ML tree with 1,000 bootstrap resamplings. T2SS, type 2 secretion system; T3SS, type 3 secretion system; T5SS, type 5 secretion system; T6SS, type 6 secretion system; AAI, adhesion and invasion; CU fimbria, chaperone-usher fimbria.

Depending on their phylogenetic background, the ABU isolates differed markedly in the presence of ExPEC virulence- and fitness-associated factors (**Figure 6, Table 3**, and **Supplementary Figure S3** and **Supplementary Table S3**). Phylogroup B2 strains, ABU as well as the model UPEC isolates, exhibited the highest prevalence of ExPEC virulence/fitness-associated gene products (**Table 3**). According to the presence/absence heatmap, phylogroup B2 and F strains could be easily distinguished from group D and B1 strains based on their virulence/fitness factor profiles. Within phylogroup B2, the ST95 and ST73 isolates could be clearly discriminated according to their virulence/fitness factor content (**Figure 6**). Similar numbers of virulence/fitness-associated gene products were predicted for ABU strains 61 (*n* = 280) and 65 (*n* = 278) as well as for ABU isolates 106 (*n* = 330) and 84 (*n* = 341). Of all strains analyzed, the highest number of virulence/fitness-associated gene products was assigned to ABU isolate 91 (*n* = 387). The lowest number (*n* = 250) of the 1,154 ExPEC virulence-associated protein sequences included into our database was detected in ABU strain 1. In ABU strains 9, 123, and 148, the database search identified 368, 307, and 322 virulence- and fitness-associated factors, respectively.

**Table 3:** Detection of ExPEC virulence/fitness-associated determinants in ABU isolates and model UPEC strains.

*E. coli* strain	Phylogroup	Total no. of predicted ExPEC virulence/fitness-associated gene products	No. of bacteriocin types	No. of CU fimbrial types	No. of iron uptake systems	No. of toxins	T6SS types
ABU 1	B1	250	0	12	4	1	T6SS-2
ABU 9	B2	368	6	9	10	6	T6SS-1, T6SS-2
ABU 61	F	280	4	8	11	3	–
ABU 65	F	278	8	9	13	1	T6SS-1
ABU 84	B2	341	5	9	11	5	T6SS-1
ABU 91	B2	387	4	9	12	6	T6SS-1, T6SS-2
ABU 106	B2	330	6	8	12	2	T6SS-1, T6SS-2
ABU 123	D	307	3	10	7	1	T6SS-2
ABU 148	B2	322	4	9	10	5	T6SS-1
83972	B2	341	5	10^∗^	11	5^#^	T6SS-1
536	B2	370	1	10	9	4	T6SS-1, T6SS-2
CFT073	B2	322	5	10	10	3	T6SS-1
UTI89	B2	360	1	9	9	5	T6SS-1, T6SS-2

*^∗^The type 1- and P-fimbrial determinants can be at least partially detected, but functional fimbrial adhesins are not expressed. ^#^α-hemolysin determinant is present, but the toxin is non-functional.*

Of the 16 microcin or colicin determinants overall detected in the ABU strains, the individual isolates carried between zero and eight different microcin/colicin-encoding gene clusters. In the model ABU strain 83972 as well as in UPEC strain CFT073 five microcin determinants were detectable, whereas in UPEC strains 536 and UTI89 only one microcin or colicin gene cluster was detected (**Figure 6** and **Table 3**). The outer membrane protein CdiB of the ‘contact-dependent inhibition of growth’ antagonistic two-partner secretion protein was present in three UPEC and the ABU model organisms as well as in the ABU isolates 9, 65, 84, and 91.

The presence of 18 different factors and systems involved in iron uptake was surveyed in our strain panel. We identified between 4 and 13 iron uptake-related factors in the genomes of the ABU isolates, but only 9 or 10 of these factors were detectable in the UPEC model strains. A total of 11 factors associated with iron uptake were found in ABU strain 83972 (**Figure 6** and **Table 3**).

We included 21 chaperone-usher (CU) pathway-dependent fimbrial determinants into our virulence/fitness factor screening. P-fimbria-related gene products were detectable in the model UPEC strains and *E. coli* 83972, as well as in ABU isolate 84, but not consistently identified in the other ABU isolates. The S-/F1C fimbrial proteins were present in the UPEC and ABU model strains, but also detectable in ABU isolates 9, 84, 91, and 148. Altogether, the number of CU fimbrial types detected in the ABU isolates varied between 8 and 12. Similarly, 10 of these adhesin types were identified in ABU model strain 83972 and UPEC strains CFT073 and 536, whereas UPEC strain UTI89 was positive for 9 members of the CU fimbria family (**Figure 6** and **Table 3**).

The ABU isolates of phylogoups B1, D, and F did not carry genes coding for the toxins α-hemolysin, cytotoxic necrotizing factor (CNF), cytolethal distending toxin (CDT), or the colibactin polyketide (Clb). With one exception (ABU strain 106), all the phylogroup B2 strains were positively tested for the aforementioned toxin determinants. Except for ABU strains 1, 61, and 123, the T6SS-1 system, which is considered to confer anti-bacterial activity (Journet and Cascales, 2016), was detected in all remaining ABU isolates. T6SS-2 determinants, supposed to be involved in host cell invasion and manipulation (Journet and Cascales, 2016), were less prevalent and only found in UPEC strains 536 and UTI89 as well as in the ABU isolates 1, 9, 91, and 106.

The virulence- and fitness gene binary matrix was used to examine the relationship of present/absent virulence- and fitness-associated genes with the strains’ phylogroup and sequence type via multivariate analyses. PCoA was plotted based on a Bray–Curtis similarity matrix of the BLASTP hits to examine the grouping of strains according to the virulence- and fitness-associated gene matrix, phylogeny, and their competitiveness in urine (**Supplementary Figure S4**). We could not observe a correlation between virulence- and fitness-associated gene content or phylogroup and competitiveness.

**FIGURE 7:**
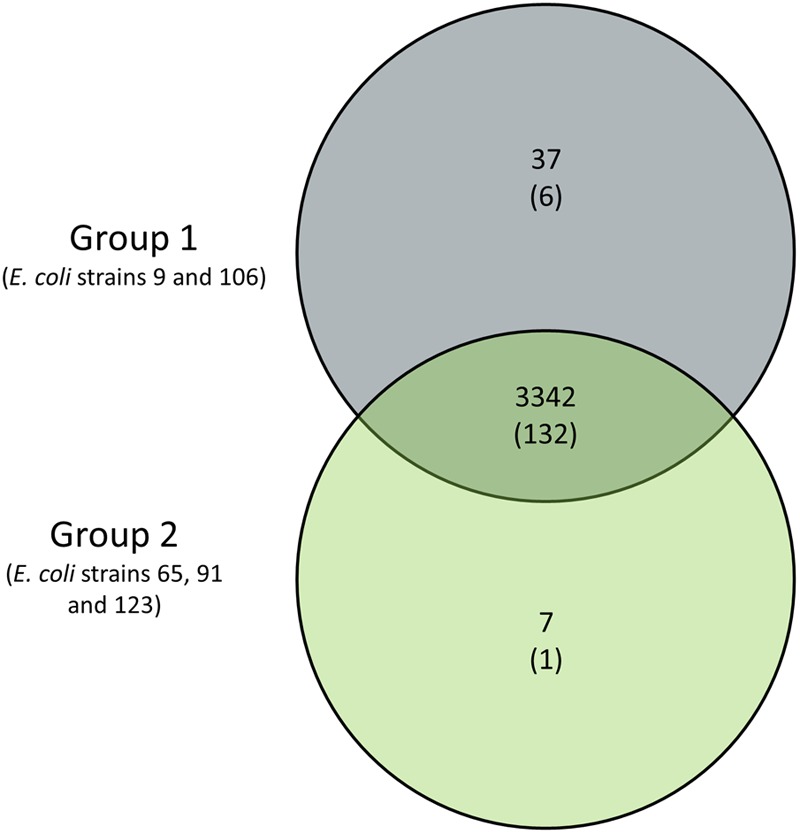
Venn diagram of genes specific for ABU strains with increased competitiveness compared to ABU model strain 83972 and/or UPEC strain 536. ABU strains 9 and 106, representing group 1, displayed superior fitness relative to model strains 83972 and 536. ABU isolates 65, 91, and 123 allocated to group 2 only outcompeted *E. coli* strain 83972. The number of group-specific ExPEC virulence- and fitness-associated gene products is given in parentheses.

The competition assay revealed that the ABU strains 9, 65, 91, 106, and 123 can outcompete prototypic ABU strain 83972 in pooled human urine within 3 h. However, only ABU strains 9 and 106 were also able to outcompete UPEC strain 536 (**Figure 4**). These two strains secreted bacteriocins, which inhibited growth of *E. coli* 83972 and UPEC 536 (**Supplementary Figure S1C**). To screen for common traits associated with the increased competitiveness of the latter two strains, we compared the genome content of two groups of isolates: Group 1 contained the ABU isolates 9 and 106 with increased fitness compared to UPEC strain 536 and ABU isolate 83972, while the second group comprised the remaining three ABU strains 65, 91, and 123 that only outcompeted *E. coli* 83972 (**Figure 7**). Since we used draft genomes, we chose non-stringent parameters of 70% identity and 70% coverage for the pairwise comparison of the presence/absence of gene products. With the used set of parameters, the shared core proteome of all five analyzed ABU isolates comprised 3,342 predicted orthologs. Of the 3,342 predicted orthologs shared between group 1 and group 2 strains, 132 proteins (3.95 %) have a virulence- and fitness-associated function (**Figure 7** and **Supplementary Tables S4, S5**) and belong to the functional categories of adhesins and invasins, T2SS, T5SS, CU fimbria, flagella/chemotaxis, as well as iron uptake and serum resistance factors (**Figure 6** and **Supplementary Table S4**). No shared proteins were found in the five ABU isolates tested for the functional categories bacteriocins, T3SS, T6SS, toxins, and type 4 pilus. Interestingly, 37 gene products were unique to ABU strains 9 and 106 (**Figure 7**). Six of them have a virulence-and fitness-associated function and represent the different proteins involved in the biosynthesis and transport of the aerobactin siderophore (IutA, IucDCBA, ShiF) (**Supplementary Table S3**). The other “group 1-specific” proteins, that have not been correlated with ExPEC virulence of fitness so far, include many prophage-encoded proteins involved in DNA repair, DNA restriction-modification, and uncharacterized proteins as well as an N-terminally truncated ZnuB Zn^2+^ ABC transporter membrane subunit (**Supplementary Table S5**). Only seven proteins appeared to be “specific” for group 2 strains, including the proteins involved in the biosynthesis of the osmoprotectant glycine betaine from choline (BetTIBA). The Zn^2+^ ABC transporter membrane subunit ZnuB is intact in the group 2 strains (**Supplementary Table S5**). The so-called “*Salmonella* iron transport (Sit)” siderophore system was completely detectable only in the group 2 isolates. Accordingly, the SitD protein was the only virulence- and fitness-related protein specifically detected in group 2 (**Figures 6, 7** and **Supplementary Table S4**).

In summary, the genomic characterization of the ABU isolates demonstrated that the majority of these urine isolates belonged to phylogroup B2, but also to other phylogenetic lineages. The phylogroup B2 isolates represented two major ExPEC clones, i.e., ST95 and ST73. Only two of the nine isolates carried antibiotic resistance determinants and exhibited resistance phenotypes. The strains’ phylogenetic background, the overall number of known ExPEC-related virulence- and fitness-associated factors as well as the prevalence of individual functional groups of ExPEC virulence- and fitness-associated factors (adhesins, iron uptake, toxins, and antagonistic factors including bacteriocins) could not be correlated with increased fitness in urine.

## Discussion

The prototypic ABU *E. coli* strain 83972 has been successfully used for the prevention or treatment of patients suffering from chronic or recurrent UTI by bacterial interference upon deliberate colonization. Bacterial interference is a promising alternative to antibiotic treatment in order to prevent bladder colonization by invading pathogens. Successful colonization with a competitive asymptomatic colonizer can confer colonization resistance to uropathogens without increasing the risk of resistance development. Bacterial interference due to deliberate colonization with *E. coli* strain 83972 is efficient and safe and has been demonstrated in placebo-controlled studies (Wullt et al., 1998; Sundén et al., 2010; Köves et al., 2014). Several characteristics of strain 83972 have been considered as important traits contributing to the competitiveness in the urinary tract, including good biofilm formation and rapid growth in urine. The expression of yersiniabactin was correlated with the capacity to efficiently form biofilms in iron-poor environments, such as the human urine (Hancock et al., 2008). *E. coli* 83972 is serum sensitive, has a rough LPS, does usually not express flagella, and lacks functional P-, type 1- and F1C fimbria as well as the functional toxin α-hemolysin (Klemm et al., 2006; Roos et al., 2006b; Zdziarski et al., 2008, 2010). Nevertheless, prototypic ABU isolate 83972 evolved from an uropathogenic ancestor and expresses the cytotoxic necrotizing factor 1 (CNF-1) as well as colibactin, two fitness factors that could also contribute to virulence. Despite the fact that there is no evidence that the prototypic ABU strain 83972 reverts to a more virulent genotype (Zdziarski et al., 2010; Köves et al., 2014), other ABU strains lacking additional VFs may pose an alternative with a lower risk of causing symptoms. Therefore, we wanted to search for ABU isolates with an even lower pathogenic potential, but similar or superior competitiveness compared to *E. coli* 83972 to offer alternative treatment options for deliberate colonization. The comparison of virulence- and fitness-related traits of *E. coli* isolates with the strains’ competitiveness in urine will also increase our fundamental understanding of bacterial characteristics relevant for efficient growth in urine as well as the safety and therapeutic success of deliberate asymptomatic bladder colonization. In this study, we geno- and phenotypically analyzed nine ABU *E. coli* isolates and compared their genome content, selected ExPEC fitness traits, and competitiveness with those of well-characterized ABU or UPEC model isolates.

*E. coli* model ABU strain 83972 has been shown to outcompete well-characterized UPEC strains, such as *E. coli* CFT073, 536, and NU14 in pooled human urine in batch cultures (Roos et al., 2006c). In the present study, six out of nine ABU isolates from diabetic patients were able to replace ABU model strain 83972 in pooled human urine (**Figure 4**). Only two of them, strains 9 and 106, also interfered with growth of model UPEC strain 536 in urine (**Figure 4B**). This suggests that these two isolates have a competitive advantage against different relevant *E. coli* strains in urine. Whereas *E. coli* 83972 was reported to outcompete *E. coli* strain 536 after 17 h of incubation under shaking conditions (Roos et al., 2006c), we observed only a minor competitive effect of *E. coli* 83972 in pooled human urine under shaking or static growth conditions in our competition experiments (**Figure 4B** and data not shown). Most likely, slight differences between the *E. coli* 83972 and/or 536 strain variants used in the different laboratories as well as in the different experimental setup are responsible for the different outcome of the competition assays.

Roos et al. (2006a) described for nine ABU *E. coli* isolates, that the frequent lack of functional fimbrial adhesins as well as an increased growth rate in urine were characteristic of many ABU isolates. Furthermore, these authors reported a positive correlation between the growth rate of ABU isolates in urine and the competition performance against UPEC strain CFT073. The frequent lack of functional adhesins such as type 1- or P-fimbria has also been reported for a larger collection of ABU isolates (Salvador et al., 2012). In the present study, only one out of nine ABU isolates was able to express type 1 fimbria, and none of them expressed P-fimbria (**Table 2**). Nevertheless, all the strains included into our study displayed very similar growth rates during the exponential growth phase, but differed in their optical density reached at the end of the experiment (**Figure 3**). Strains with increased competitiveness against *E. coli* 83972 did not consistently display lack of functional type 1 fimbria or superior growth in urine. The three ABU isolates 1, 84, and 148 which could not replace *E. coli* strain 83972 in urine displayed lower final optical densities relative to the latter isolate. Even the strain with the poorest growth behavior in urine, ABU strain 61, was able to significantly reduce the fraction of *E. coli* 83972 cells in the mixed culture (**Figure 4A**). Accordingly, the idea that the competitiveness against UPEC or other bacteria colonizing the bladder depends more or less on the strain’s ability to grow fast in urine as coined by Roos et al. (2006a) is controversial.

Colicin production has been reported to interfere with the colonization of catheters by colicin-sensitive *E. coli* variants (Trautner et al., 2005) and a higher frequency of bacteriocin expression was found in ExPEC and phylogroup B2 strains relative to fecal isolates (Micenková et al., 2016a,b). Consequently, secretion of inhibitory molecules as well as expression of other antagonistic traits, such as the contact-dependent inhibition of growth system (CdiAB), together with the resistance against antagonistic factors increase the competitiveness of *E. coli*. The results of the bacteriocin agar plate assays (**Supplementary Figure S1**) suggest that the expression of secreted inhibitory compounds contributes to the increased competitiveness of ABU isolates 9 and 106 against UPEC strain 536 and ABU model strain 83972. Four additional ABU isolates also secreted bacteriocins or other inhibitors, which interfered with growth of *E. coli* strain DH5α. Altogether, 16 different determinants coding for microcins or colicins could be detected in the genomes of the ABU isolates (**Figure 6** and **Table 3**). The number of detected operons or the type of antagonistic factor could, however, not be correlated with increased competitiveness against *E. coli* strains 83972 or 536 (**Table 3, Supplementary Figure S4**, and **Supplementary Table S3**).

Growth of UPEC in urine depends on the availability of iron, zinc, and manganese ions (Sabri et al., 2009; Porcheron et al., 2013) and thus it is not surprising that UPEC strains encode multiple siderophore systems and that siderophore expression is upregulated in urine (Snyder et al., 2004; Hagan et al., 2010; Zdziarski et al., 2010). Whereas enterobactin may not be important for growth in urine, the expression of yersiniabactin and salmochelin seems to be more important for iron uptake during growth in urine (Henderson et al., 2009). Interestingly, general siderophore secretion on CAS agar plates did not correlate with increased competitiveness in urine (**Supplementary Figure S1B**). Similarly, the overall number of determinants coding for factors involved in iron acquisition as detected by comparative genomics did not correlate with the competitiveness of the strains relative to ABU strain 83927 (**Table 3, Supplementary Figure S4**, and **Supplementary Table S3**). The fact, however, that the ABU isolates 9 and 106 (group 1), which outcompeted *E. coli* strains 83972 and 536, share the aerobactin determinant in comparison with strains 65, 91, 123 (group 2), indicates that the expression of this siderophore system may directly or indirectly contribute to increased fitness and competitiveness in urine. The ZnuACB ABC transport system required for the uptake of zinc and several other metal cations during growth in urine (Sabri et al., 2009) was detectable in both groups of strains, though with structural differences.

Among the strains tested, the majority of the ABU isolates including the two strains with the highest competitiveness in urine belong to phylogenetic lineage B2. This is the phylogenetic lineage, which usually comprises *E. coli* isolates with a higher potential to cause extraintestinal infection (Tenaillon et al., 2010), but phylogroup B2 strains have also been described to efficiently colonize the intestinal tract (Nowrouzian et al., 2005, 2006). So-called “ExPEC” virulence traits have not primarily evolved to contribute to extraintestinal infection, but rather to increase the fitness of *E. coli* in its natural niche, i.e., the intestinal tract (Nowrouzian et al., 2001; Ostblom et al., 2011). Obviously, such ExPEC virulence-associated traits may also support bacterial fitness and competitiveness in urine. Previous studies report that an increased number of gene clusters coding for ExPEC virulence-associated traits contributes to the increased fitness of B2 strains compared to members of other *E. coli* phylogroups in the intestinal tract (Nowrouzian et al., 2005, 2006, 2009; Ostblom et al., 2011; Smati et al., 2017). This indicates that there is a relation between the accumulation of ExPEC virulence-associated factors and the persistence of *E. coli* within the intestine (Ostblom et al., 2011), but also in urine. Studies on within-host competition of different *E. coli* strains in the intestinal tract showed that the presence of phylogroup B2 strains seems to inhibit the establishment of strains belonging to other phylogroups (Blyton et al., 2014; Gordon et al., 2015; Smati et al., 2017). Transferring these findings to growth in urine, it is therefore not surprising that many of the ABU isolates in our study belong to phylogroup B2 and that several of them exhibit increased competitiveness relative to the ABU model strain 83972. For *E. coli* strains colonizing the colon, previous studies reported that resident strains carry more often genes encoding the siderophore system aerobactin compared to transient strains (Nowrouzian et al., 2001), suggesting that this siderophore system directly or indirectly confers a competitive advantage. This may also explain the successful displacement of *E. coli* strains 83972 and 536 from mixed urine cultures by aerobactin-positive ABU isolates 9 and 106. Similarly, a correlation between gene content, including virulence-associated genes, and virulence (as determined in selected *in vivo* models) could not be identified for uropathogenic *E. coli* and bacteremia *E. coli* isolates (Landraud et al., 2013; Schreiber et al., 2017). Consequently, differential expression of (conserved) genes in response to the growth conditions encountered *in vivo* may determine the competitiveness and fitness of *E. coli* isolates.

We are aware of the limitation of our study that only two model strains, i.e., ABU strain 83972 and UPEC strain 536, have been used as competitors in the growth competition assays. Given the high variability of *E. coli*, future studies should include additional UPEC isolates with different phylogenetic backgrounds and different genome content. The success of deliberate therapeutic colonization will be even higher if such competitor strains would not only be able to prevent *E. coli* strains from infecting the bladder, but also other uropathogens. Competitiveness of the ABU *E. coli* isolates should therefore be tested against different isolates from other relevant enterobacterial species including *Proteus mirabilis* and *K. pneumoniae*, but maybe also against uropathogens, which are frequently isolated from complicated UTI and from hospitalized patients such as *Staphylococcus saprophyticus, Enterococcus faecalis*, and *Pseudomonas aeruginosa*. Competitiveness and long-term survival in the bladder (including formation of intracellular bacterial communities) of the candidate strains for bacterial interference should be tested *in vitro*, but also in suitable *in vivo* infection models. *E. coli* is characterized by tremendous genome plasticity, and many ABU *E. coli* isolates evolved by reductive evolution from uropathogenic ancestors (Zdziarski et al., 2008, 2010). Consequently, the risk of regaining uropathogenic potential by acquisition of virulence-associated genes from other bacteria via horizontal gene transfer or genomic rearrangements is a concern raised in connection with deliberate colonization. Therapeutic colonization with *E. coli* strain 83972 has been proven to be safe (Köves et al., 2014). Yet, the general acceptance of this promising alternative to antibiotic treatment will further benefit from a more detailed understanding of the interfering strains’ interaction with and sensing by the host immune system that could result in the onset of symptoms of inflammation. Microevolution of ABU isolates during deliberate colonization is also an important aspect that requires thorough investigation.

Our search for safe and efficient ABU isolates for preventive or therapeutic bladder colonization aimed at the identification of strains with a low potential to cause extraintestinal infection, but high fitness and competitiveness in urine. Optimally, suitable *E. coli* strains should be highly competitive in urine, but at the same time as non-virulent and antibiotic susceptible as possible. Our analysis confirms previous findings that increased fitness in urine correlates with the presence of ExPEC virulence-associated factors. Six ABU isolates exhibited superior fitness relative to ABU model strain 83972, and two of them also outcompeted UPEC strain 536 in pooled human urine. These strains belong to different phylogenetic lineages and sequence types and differ markedly in their ability to express ExPEC virulence-associated factors. Two ABU isolates with superior competitiveness compared to *E. coli* 83972 and/or UPEC 536, *E. coli* strains 9 and 91, exhibited a marked hemolytic and cytotoxic potential as well as serum resistance and had low LD50 values in murine sepsis model. Accordingly, these two strains, although they efficiently displaced ABU model strain 83972 from mixed urine cultures, are less favorable candidates to be further included into the development of improved therapeutic bladder colonization strategies. In contrast, ABU strain 65 outcompeted *E. coli* 83972 in urine and exhibited a very high LD50 value in the sepsis model, but lacked typical ExPEC virulence traits. This ABU strain may be a promising candidate for efficient therapeutic bacterial interference. The fact that this strain is resistant to aminoglycosides and β-lactams poses a risk to the patients. The antibiotic resistance of ABU strain 65 is Janus-faced from the point of view of being a competitive and good colonizer of the urinary tract with a low virulence potential, but being less liable for eradication by antibiotic treatment than other strains. This could, nevertheless also be an advantage if a patient has to be treated with antibiotics for other reasons as this strain may survive the treatment in the bladder. Its resistance pattern is known, and can be considered if antibiotic treatment of other infections may be necessary. Deliberate therapeutic bladder colonization could thus be unaffected even during treatment with selected antibiotics. The remaining ABU isolates 61, 106, and 123, however, are the most promising alternative strains to be used for bacterial interference in the urinary bladder. They are characterized by low *in vivo* virulence in the murine sepsis model, susceptibility to antibiotics and increased competitiveness in urine relative to *E. coli* 83972. They belong to different phylogroups and thus differ in the presence of ExPEC virulence- and fitness-associated genes. Importantly, they all lack marked cytotoxic activity and exhibited a high LD50 value in the sepsis model. They differ, however, in their repertoire of ExPEC fitness-related factors, which may promote their competitiveness. Growth in urine also requires efficient uptake of nutrients, and metabolic traits promote urovirulence of *E. coli* (Nielsen et al., 2017; Schreiber et al., 2017). Mutants that lack the ability to synthesize different amino acids exhibit growth defects in urine (Hull and Hull, 1997). The expression of genes involved in amino acid and carbohydrate metabolism has been shown to be upregulated upon growth in urine (Snyder et al., 2004; Hagan et al., 2010; Zdziarski et al., 2010). Increased fitness of UPEC correlates with efficient growth on dipeptides and several glucogenic amino acids (Chen et al., 2013). Consequently, the metabolic potential of the ABU isolates will markedly contribute to the fitness and thus competitiveness in urine and it will be interesting to analyze in future studies the metabolic potential and individual gene expression pattern of the isolates in relation to their fitness in urine. Accordingly, future studies will be required to characterize in-depth the molecular basis of their marked competitiveness in urine.

## Author Contributions

LE and UD designed this project. CS, BK, BR, JP, MK, ÁD, JK, SM, AL, TK, GS, and MK contributed to the collection of ABU isolates, the experiments, data analysis, and interpretation of the results. CS, LE, and UD drafted the manuscript. All authors critically revised and approved the manuscript.

## Conflict of Interest Statement

The authors declare that the research was conducted in the absence of any commercial or financial relationships that could be construed as a potential conflict of interest.

## Footnotes

1 https://github.com/dobrindtlab/shell_scripts

2 https://github.com/dobrindtlab/gene_analysis

## Supplementary Material

FIGURE S1 Phenotypic traits of nine ABU isolates. **(A)** Hemolysin production on Columbia blood agar. **(B)** Siderophore production on CAS agar. **(C)** Bacteriocin production tested with different indicator strains, i.e. *E. coli* DH5α, UPEC 536, ABU isolate 83972. **(D)** Expression of rdar morphotype, i.e. simultaneous expression of curli fimbria and cellulose on Congo Red agar plates at 30 °C or 37 °C.

FIGURE S2 Competitive growth experiments between nine *E. coli* ABU isolates and ABU model isolate *E. coli* 83972 in pooled human urine. The results of competitive growth experiments (mixed cultures, 1:1) between the ABU isolates and ABU model strain 83972 after 24 h of growth in pooled human urine are indicated. Asterisks indicate significantly different competitive fitness compared to reference strains 83972 (paired two-tailed *t*-test; ^∗^, *P* < 0.05; ^∗∗^, *P* < 0.005; ^∗∗∗^, *P* < 0.0005; ^∗∗∗∗^, *P* < 0.0001). The data shown in the graph are mean values, the error bars indicate SD.

FIGURE S3 Heatmap indicating the presence or absence of ExPEC virulence- and fitness-associated factors including gene names/locus tags. Each row of the binary matrix indicates the presence or absence of a virulence/fitness-associated gene product (a BLASTP+ hit). Virulence/fitness factor classes are indicated at the side in black and grey. Strain names and individual columns are color-coded for ABU isolates (green) or UPEC strains (blue). Well-known ExPEC virulence determinants are indicated by red boxes. The clustering dendrogram attached to the heatmaps is based on the whole binary dataset of a best scoring ML tree with 1000 bootstrap resamplings. T2SS, type 2 secretion system; T3SS, type 3 secretion system; T5SS, type 5 secretion system; T6SS, type 6 secretion system; AAI, adhesion and invasion; CU fimbria, chaperone-usher fimbria.

FIGURE S4 Principal Coordinates Analysis (PCoA) to examine the grouping of *E. coli* ABU isolates according to the presence/absence of fitness- and virulence-associated genes and their competitiveness in urine. The axes are scaled with eigenvalue scaling using the square root of the eigenvalue and indicate the percentage of variation explained in the PCoA. Strains of the same sequence type (ST) are indicated by the same symbol. Symbols are colored according to the phylogroup of each strain. Additionally, superior competitiveness of ABU isolates to model ABU strain 83972 and UPEC strain 563 is indicated.

TABLE S1 Bacterial strains used in this study.

TABLE S2 Characteristics of the patients.

TABLE S3 Presence or absence of 616 ExPEC virulence/fitness-associated gene products in the ABU and model UPEC strains.

TABLE S4 Strain- and group-specific virulence/fitness-associated factors detected in group 1 or 2 ABU strains with increased competitiveness to *E. coli* 83972 and/or UPEC strain 536.

TABLE S5 Strain- and group-specific genes detected in group 1 or 2 ABU strains with increased competitiveness to *E. coli* 83972 and/or UPEC strain 536.

## References

[B1] Andrews S. (2010). *FastQC: A Quality Control Tool for High Throughput Sequence Data.* Available at: http://www.bioinformatics.babraham.ac.uk/projects/fastqc

[B2] Bankevich A. Nurk S. Antipov D. Gurevich A. A. Dvorkin M. Kulikov A. S. (2012). SPAdes: a new genome assembly algorithm and its applications to single-cell sequencing. *J. Comput. Biol.* 19 455–477. 10.1089/cmb.2012.0021 22506599 PMC3342519

[B3] Blyton M. D. Cornall S. J. Kennedy K. Colligon P. Gordon D. M. (2014). Sex-dependent competitive dominance of phylogenetic group B2 *Escherichia coli* strains within human hosts. *Environ. Microbiol. Rep.* 6 605–610. 10.1111/1758-2229.12168 25756113

[B4] Cai T. Köves B. Johansen T. E. (2017). Asymptomatic bacteriuria, to screen or not to screen - and when to treat? *Curr. Opin. Urol.* 27 107–111. 10.1097/MOU.0000000000000368 27906777

[B5] Carattoli A. Zankari E. García-Fernández A. Voldby Larsen M. Lund O. Villa L. (2014). *In silico* detection and typing of plasmids using PlasmidFinder and plasmid multilocus sequence typing. *Antimicrob. Agents Chemother.* 58 3895–3903. 10.1128/AAC.02412-14 24777092 PMC4068535

[B6] Cassini A. Plachouras D. Eckmanns T. Abu Sin M. Blank H. P. Ducomble T. (2016). Burden of six healthcare-associated infections on European population health: estimating incidence-based disability-adjusted life years through a population prevalence-based modelling study. *PLOS Med.* 13:e1002150. 10.1371/journal.pmed.1002150 27755545 PMC5068791

[B7] Chen S. L. Wu M. Henderson J. P. Hooton T. M. Hibbing M. E. Hultgren S. J. (2013). Genomic diversity and fitness of *E. coli* strains recovered from the intestinal and urinary tracts of women with recurrent urinary tract infection. *Sci. Transl. Med.* 5:184ra160. 10.1126/scitranslmed.3005497 23658245 PMC3695744

[B8] Darouiche R. O. Green B. G. Donovan W. H. Chen D. Schwartz M. Merritt J. (2011). Multicenter randomized controlled trial of bacterial interference for prevention of urinary tract infection in patients with neurogenic bladder. *Urology* 78 341–346. 10.1016/j.urology.2011.03.062 21683991

[B9] Darouiche R. O. Hull R. A. (2012). Bacterial interference for prevention of urinary tract infection. *Clin. Infect. Dis.* 55 1400–1407. 10.1093/cid/cis639 22828592

[B10] Flores-Mireles A. L. Walker J. N. Caparon M. Hultgren S. J. (2015). Urinary tract infections: epidemiology, mechanisms of infection and treatment options. *Nat. Rev. Microbiol.* 13 269–284. 10.1038/nrmicro3432 25853778 PMC4457377

[B11] Foxman B. (2014). Urinary tract infection syndromes: occurrence, recurrence, bacteriology, risk factors, and disease burden. *Infect. Dis. Clin. North Am.* 28 1–13. 10.1016/j.idc.2013.09.003 24484571

[B12] Fredericq P. (1957). Colicins. *Ann. Rev. Microbiol.* 11 7–22. 10.1146/annurev.mi.11.100157.000255 13470810

[B13] Gordon D. M. O’Brien C. L. Pavli P. (2015). *Escherichia coli* diversity in the lower intestinal tract of humans. *Environ. Microbiol. Rep.* 7 642–648. 10.1111/1758-2229.12300 26034010

[B14] Grabe M. Bartoletti R. Bjerklund Johansen T. E. Cai T. Çek M. Köves B. (2015). *Guidelines on Urological Infections.* Arnhem: European Association of Urology.

[B15] Gurevich A. Saveliev V. Vyahhi N. Tesler G. (2013). QUAST: quality assessment tool for genome assemblies. *Bioinformatics* 29 1072–1075. 10.1093/bioinformatics/btt086 23422339 PMC3624806

[B16] Hacker J. Hof H. Emödy L. Goebel W. (1986). Influence of cloned *Escherichia coli* hemolysin genes, S-fimbriae and serum resistance on pathogenicity in different animal models. *Microb Pathogen.* 1 533–547. 10.1016/0882-4010(86)90039-2 2907773

[B17] Hagan E. C. Lloyd A. L. Rasko D. A. Faerber G. J. Mobley H. L. (2010). *Escherichia coli* global gene expression in urine from women with urinary tract infection. *PLOS Pathog.* 6:e1001187. 10.1371/journal.ppat.1001187 21085611 PMC2978726

[B18] Hammer O. Harper D. A. T. Ryan P. D. (2001). PAST: paleontological statistics soft-ware package for education and data analysis. *Palaeontol. Electron.* 4:9.

[B19] Hancock V. Ferrieres L. Klemm P. (2008). The ferric yersiniabactin uptake receptor FyuA is required for efficient biofilm formation by urinary tract infectious *Escherichia coli* in human urine. *Microbiology* 154 167–175. 10.1099/mic.0.2007/011981-0 18174135

[B20] Henderson J. P. Crowley J. R. Pinkner J. S. Walker J. N. Tsukayama P. Stamm W. E. (2009). Quantitative metabolomics reveals an epigenetic blueprint for iron acquisition in uropathogenic *Escherichia coli*. *PLOS Pathog.* 5:e1000305. 10.1371/journal.ppat.1000305 19229321 PMC2637984

[B21] Hull R. A. Hull S. I. (1997). Nutritional requirements for growth of uropathogenic *Escherichia coli* in human urine. *Infect. Immun.* 65 1960–1961. 9125589 10.1128/iai.65.5.1960-1961.1997 PMC175252

[B22] Hull R. A. Rudy D. Donovan W. Svanborg C. Wieser I. Stewart C. (2000). Urinary tract infection prophylaxis using *Escherichia coli* 83972 in spinal cord injured patients. *J. Urol.* 163 872–877. 10687996

[B23] Jarvis W. R. (1996). Selected aspects of the socioeconomic impact of nosocomial infections: morbidity, mortality, cost, and prevention. *Infect. Control Hosp. Epidemiol.* 17 552–557. 10.2307/30141291 8875302

[B24] Joensen K. G. Tetzschner A. M. Iguchi A. Aarestrup F. M. Scheutz F. (2015). Rapid and easy *in silico* serotyping of *Escherichia coli* isolates by use of whole-genome sequencing data. *J. Clin. Microbiol.* 53 2410–2426. 10.1128/JCM.00008-15 25972421 PMC4508402

[B25] Joshi N. A. Fass J. N. (2011). *Sickle: A Sliding-Window, Adaptive, Quality-Based Trimming Tool for FASTQ Files.* Available at: https://Github.com/najoshi/sickle

[B26] Journet L. Cascales E. (2016). The type VI secretion system in *Escherichia coli* and related species. *EcoSal Plus* 7 10.1128/ecosalplus.ESP-0009-2015 27223818 PMC11575709

[B27] Kienle Z. Emödy L. Svanborg C. O’Toole P. W. (1992). Adhesive properties conferred by the plasminogen activator of *Yersinia pestis*. *J. Gen. Microbiol.* 138 1679–1687. 10.1099/00221287-138-8-1679 1527508

[B28] Klemm P. Roos V. Ulett G. C. Svanborg C. Schembri M. A. (2006). Molecular characterization of the *Escherichia coli* asymptomatic bacteriuria strain 83972: the taming of a pathogen. *Infect Immun.* 74 781–785. 10.1128/IAI.74.1.781-785.2006 16369040 PMC1346676

[B29] Köves B. Salvador E. Grönberg-Hernández J. Zdziarski J. Wullt B. Svanborg C. (2014). Rare emergence of symptoms during long-term asymptomatic *Escherichia coli* 83972 carriage without an altered virulence factor repertoire. *J. Urol.* 191 519–528. 10.1016/j.juro.2013.07.060 23911604

[B30] Kuch B. Pál T. Emödy L. Schwartz J. Menoret P. Begue P. (1982). Bacterial adherence and urinary tract infection. *Lancet* 320 107–108. 10.1016/S0140-6736(82)91732-9

[B31] Landraud L. Jaureguy F. Frapy E. Guigon G. Gouriou S. Carbonnelle E. (2013). Severity of *Escherichia coli* bacteraemia is independent of the intrinsic virulence of the strains assessed in a mouse model. *Clin. Microbiol. Infect.* 19 85–90. 10.1111/j.1469-0691.2011.03750.x 22268649

[B32] Lechner M. Findeiss S. Steiner L. Marz M. Stadler P. F. Prohaska S. J. (2011). Proteinortho: detection of (co-)orthologs in large-scale analysis. *BMC Bioinformatics* 12:124. 10.1186/1471-2105-12-124 21526987 PMC3114741

[B33] Legendre P. Legendre L. (1998). *Numerical Ecology.* Amsterdam: Elsevier.

[B34] Leimbach A. (2016a). *Bac-Genomics-Scripts: Bovine *E. coli* Mastitis Comparative Genomics Edition.* Available at: http://dx.doi.org/10.5281/zenodo.215824

[B35] Leimbach A. (2016b). *ecoli_VF_Collection.* Available at: http://dx.doi.org/10.5281/zenodo.56686

[B36] Micenková L. Bosák J. Štaudova B. Kohoutová D. Čejková D. Woznicová V. (2016a). Microcin determinants are associated with B2 phylogroup of human fecal *Escherichia coli* isolates. *MicrobiologyOpen* 5 490–498. 10.1002/mbo3.345 26987297 PMC4906000

[B37] Micenková L. Bosák J. Vrba M. Ševčíková A. Šmajs D. (2016b). Human extraintestinal pathogenic *Escherichia coli* strains differ in prevalence of virulence factors, phylogroups, and bacteriocin determinants. *BMC Microbiol.* 16:218. 10.1186/s12866-016-0835-z 27646192 PMC5028950

[B38] Nicolle L. E. Zhanel G. G. Harding G. K. (2006). Microbiological outcomes in women with diabetes and untreated asymptomatic bacteriuria. *World J. Urol.* 24 61–65. 10.1007/s00345-005-0042-2 16389540

[B39] Nielsen K. L. Stegger M. Kiil K. Godfrey P. A. Feldgarden M. Lilje B. (2017). Whole-genome comparison of urinary pathogenic *Escherichia coli* and faecal isolates of UTI patients and healthy controls. *Int. J. Med. Microbiol.* 307 497–507. 10.1016/j.ijmm.2017.09.007 29031453 PMC5792705

[B40] Nowrouzian F. Adlerberth I. Wold A. E. (2001). P fimbriae, capsule and aerobactin characterize colonic resident *Escherichia coli*. *Epidemiol. Infect.* 126 11–18. 10.1017/S0950268801005118 11293669 PMC2869660

[B41] Nowrouzian F. L. Adlerberth I. Wold A. E. (2006). Enhanced persistence in the colonic microbiota of *Escherichia coli* strains belonging to phylogenetic group B2: role of virulence factors and adherence to colonic cells. *Microbes Infect.* 8 834–840. 10.1016/j.micinf.2005.10.011 16483819

[B42] Nowrouzian F. L. Ostblom A. E. Wold A. E. Adlerberth I. (2009). Phylogenetic group B2 *Escherichia coli* strains from the bowel microbiota of Pakistani infants carry few virulence genes and lack the capacity for long-term persistence. *Clin. Microbiol. Infect.* 15 466–472. 10.1111/j.1469-0691.2009.02706.x 19260873

[B43] Nowrouzian F. L. Wold A. E. Adlerberth I. (2005). *Escherichia coli* strains belonging to phylogenetic group B2 have superior capacity to persist in the intestinal microflora of infants. *J. Infect. Dis.* 191 1078–1083. 10.1086/427996 15747243

[B44] Ostblom A. Adlerberth I. Wold A. E. Nowrouzian F. L. (2011). Pathogenicity island markers, virulence determinants *malX* and *usp*, and the capacity of *Escherichia coli* to persist in infants’ commensal microbiotas. *Appl. Environ. Microbiol.* 77 2303–2308. 10.1128/AEM.02405-10 21317254 PMC3067437

[B45] Page A. J. Cummins C. A. Hunt M. Wong V. K. Reuter S. Holden M. T. (2015). Roary: rapid large-scale prokaryote pan genome analysis. *Bioinformatics* 31 3691–3693. 10.1093/bioinformatics/btv421 26198102 PMC4817141

[B46] Porcheron G. Garénaux A. Proulx J. Sabri M. Dozois C. M. (2013). Iron, copper, zinc, and manganese transport and regulation in pathogenic Enterobacteria: correlations between strains, site of infection and the relative importance of the different metal transport systems for virulence. *Front. Cell. Infect. Microbiol.* 3:90. 10.3389/fcimb.2013.00090 24367764 PMC3852070

[B47] Quinn G. P. Keough M. J. (2002). *Experimental Design and Data Analysis for Biologists.* Cambridge: Cambridge University Press.

[B48] R Core Team (2017). *R: A Language and Environment for Statistical Computing.* Available at: https://www.R-project.org

[B49] Ramette A. (2007). Multivariate analyses in microbial ecology. *FEMS Microbiol. Ecol.* 62 142–160. 10.1111/j.1574-6941.2007.00375.x 17892477 PMC2121141

[B50] Römling U. Sierralta W. D. Eriksson K. Normark S. (1998). Multicellular and aggregative behaviour of *Salmonella typhimurium* strains is controlled by mutations in the *agfD* promoter. *Mol. Microbiol.* 28 249–264. 10.1046/j.1365-2958.1998.00791.x 9622351

[B51] Roos V. Nielsen E. M. Klemm P. (2006a). Asymptomatic bacteriuria *Escherichia coli* strains: adhesins, growth and competition. *FEMS Microbiol. Lett.* 262 22–30. 10.1111/j.1574-6968.2006.00355.x 16907735

[B52] Roos V. Schembri M. A. Ulett G. C. Klemm P. (2006b). Asymptomatic bacteriuria *Escherichia coli* strain 83972 carries mutations in the *foc* locus and is unable to express F1C fimbriae. *Microbiology* 152 1799–1806. 10.1099/mic.0.28711-0 16735742

[B53] Roos V. Ulett G. C. Schembri M. A. Klemm P. (2006c). The asymptomatic bacteriuria *Escherichia coli* strain 83972 outcompetes uropathogenic *E. coli* strains in human urine. *Infect. Immun.* 74 615–624. 10.1128/IAI.74.1.615-624.2006 16369018 PMC1346649

[B54] Rudick C. N. Taylor A. K. Yaggie R. E. Schaeffer A. J. Klumpp D. J. (2014). Asymptomatic bacteriuria *Escherichia coli* are live biotherapeutics for UTI. *PLOS ONE* 9:e109321. 10.1371/journal.pone.0109321 25405579 PMC4236008

[B55] Russo T. A. Johnson J. R. (2003). Medical and economic impact of extraintestinal infections due to *Escherichia coli*: focus on an increasingly important endemic problem. *Microbes Infect.* 5 449–456. 10.1016/S1286-4579(03)00049-2 12738001

[B56] Sabri M. Houle S. Dozois C. M. (2009). Roles of the extraintestinal pathogenic *Escherichia coli* ZnuACB and ZupT zinc transporters during urinary tract infection. *Infect. Immun.* 77 1155–1164. 10.1128/IAI.01082-08 19103764 PMC2643633

[B57] Salvador E. Wagenlehner F. Köhler C. D. Mellmann A. Hacker J. Svanborg C. (2012). Comparison of asymptomatic bacteriuria *Escherichia coli* isolates from healthy individuals versus those from hospital patients shows that long-term bladder colonization selects for attenuated virulence phenotypes. *Infect. Immun.* 80 668–678. 10.1128/IAI.06191-11 22104113 PMC3264318

[B58] Sambrook J. Fritsch E. F. Maniatis T. (1989). *Molecular Cloning: A Laboratory Manual.* Cold Spring Harbor, NY: Cold Spring Harbor Laboratory Press.

[B59] Schreiber H. L. T. Conover M. S. Chou W. C. Hibbing M. E. Manson A. L. Dodson K. W. (2017). Bacterial virulence phenotypes of *Escherichia coli* and host susceptibility determine risk for urinary tract infections. *Sci. Transl. Med.* 9:eaaf1283. 10.1126/scitranslmed.aaf1283 28330863 PMC5653229

[B60] Schwyn B. Neilands J. B. (1987). Universal chemical assay for the detection and determination of siderophores. *Anal. Biochem.* 160 47–56. 10.1016/0003-2697(87)90612-9 2952030

[B61] Seemann T. (2014). Prokka: rapid prokaryotic genome annotation. *Bioinformatics* 30 2068–2069. 10.1093/bioinformatics/btu153 24642063

[B62] Smati M. Magistro G. Adiba S. Wieser A. Picard B. Schubert S. (2017). Strain-specific impact of the high-pathogenicity island on virulence in extra-intestinal pathogenic *Escherichia coli*. *Int. J. Med. Microbiol.* 307 44–56. 10.1016/j.ijmm.2016.11.004 27923724

[B63] Snyder J. A. Haugen B. J. Buckles E. L. Lockatell C. V. Johnson D. E. Donnenberg M. S. (2004). Transcriptome of uropathogenic *Escherichia coli* during urinary tract infection. *Infect. Immun.* 72 6373–6381. 10.1128/IAI.72.11.6373-6381.2004 15501767 PMC523057

[B64] Stamatakis A. (2014). RAxML version 8: a tool for phylogenetic analysis and post-analysis of large phylogenies. *Bioinformatics* 30 1312–1313. 10.1093/bioinformatics/btu033 24451623 PMC3998144

[B65] Stork C. Kovács B. Trost E. Kovács T. Schneider G. Rózsai B. (2018). Whole-genome draft sequences of nine asymptomatic *Escherichia coli* bacteriuria isolates from diabetic patients. *Genome Announc.* 6 e1369-17. 10.1128/genomeA.01369-17 29326206 PMC5764930

[B66] Sundén F. Håkansson L. Ljunggren E. Wullt B. (2010). *Escherichia coli* 83972 bacteriuria protects against recurrent lower urinary tract infections in patients with incomplete bladder emptying. *J. Urol.* 184 179–185. 10.1016/j.juro.2010.03.024 20483149

[B67] Tenaillon O. Skurnik D. Picard B. Denamur E. (2010). The population genetics of commensal *Escherichia coli*. *Nat. Rev. Microbiol.* 8 207–217. 10.1038/nrmicro2298 20157339

[B68] Trautner B. W. Hull R. A. Darouiche R. O. (2005). Colicins prevent colonization of urinary catheters. *J. Antimicrob. Chemother.* 56 413–415. 10.1093/jac/dki228 15980093 PMC2077848

[B69] Wullt B. Connell H. Röllano P. Månsson W. Colleen S. Svanborg C. (1998). Urodynamic factors influence the duration of *Escherichia coli* bacteriuria in deliberately colonized cases. *J. Urol.* 159 2057–2062. 10.1016/S0022-5347(01)63246-4 9598517

[B70] Zankari E. Hasman H. Cosentino S. Vestergaard M. Rasmussen S. Lund O. (2012). Identification of acquired antimicrobial resistance genes. *J. Antimicrob. Chemother.* 67 2640–2644. 10.1093/jac/dks261 22782487 PMC3468078

[B71] Zdziarski J. Brzuszkiewicz E. Wullt B. Liesegang H. Biran D. Voigt B. (2010). Host imprints on bacterial genomes - rapid, divergent evolution in individual patients. *PLOS Pathog.* 6:e1001078. 10.1371/journal.ppat.1001078 20865122 PMC2928814

[B72] Zdziarski J. Svanborg C. Wullt B. Hacker J. Dobrindt U. (2008). Molecular basis of commensalism in the urinary tract: low virulence or virulence attenuation? *Infect. Immun.* 76 695–703. 10.1128/IAI.01215-07 18039831 PMC2223460

