# Supplementary figures and images for: Characterization of Asymptomatic Bacteriuria Escherichia coli Isolates in Search of Alternative Strains for Efficient Bacterial Interference against Uropathogens
Source: Front Microbiol. 2018 Feb 14;9:214. doi: 10.3389/fmicb.2018.00214 (PMC5817090; doi:10.3389/fmicb.2018.00214)

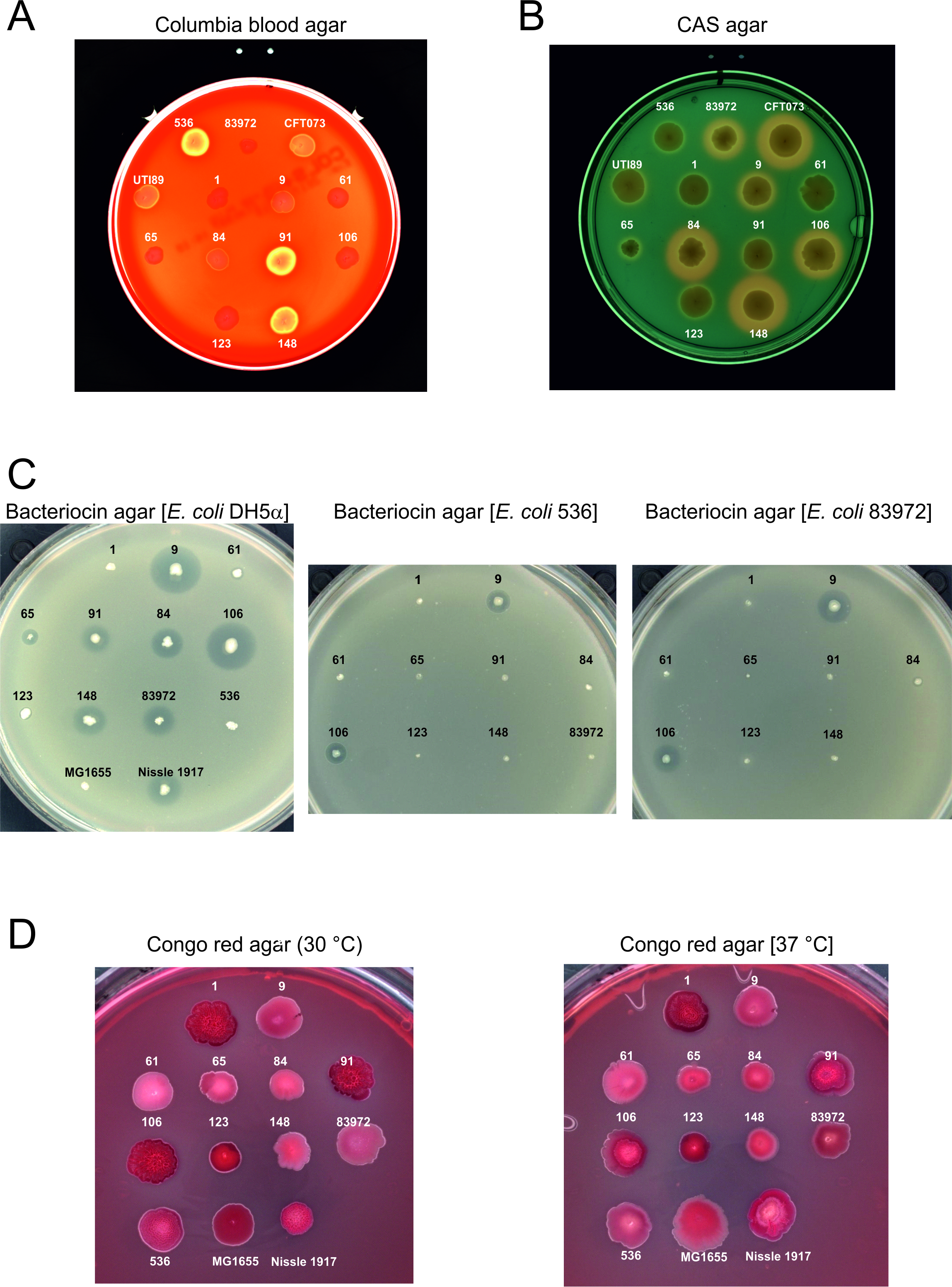

Supplement: FIGURE S1 — Phenotypic traits of nine ABU isolates. (A) Hemolysin production on Columbia blood agar. (B) Siderophore production on CAS agar. (C) Bacteriocin production tested with different indicator strains, i.e. E. coli DH5α, UPEC 536, ABU isolate 83972. (D) Expression of rdar morphotype, i.e. simultaneous expression of curli fimbria and cellulose on Congo Red agar plates at 30 °C or 37 °C. [file Image_1.JPEG]

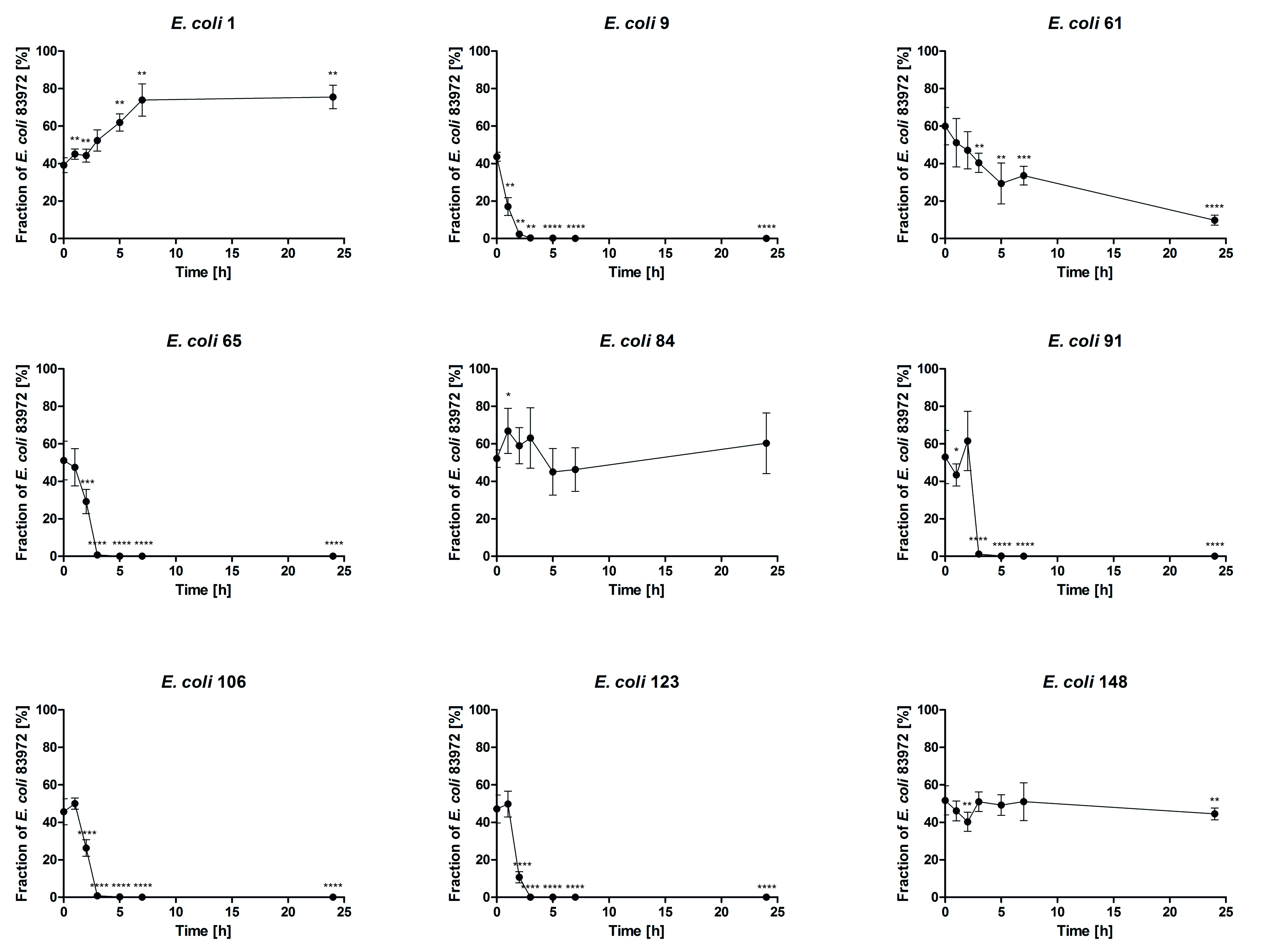

Supplement: FIGURE S2 — Competitive growth experiments between nine E. coli ABU isolates and ABU model isolate E. coli 83972 in pooled human urine. The results of competitive growth experiments (mixed cultures, 1:1) between the ABU isolates and ABU model strain 83972 after 24 h of growth in pooled human urine are indicated. Asterisks indicate significantly different competitive fitness compared to reference strains 83972 (paired two-tailed t-test; ∗, P < 0.05; ∗∗, P < 0.005; ∗∗∗, P < 0.0005; ∗∗∗∗, P < 0.0001). The data shown in the graph are mean values, the error bars indicate SD. [file Image_2.JPEG]

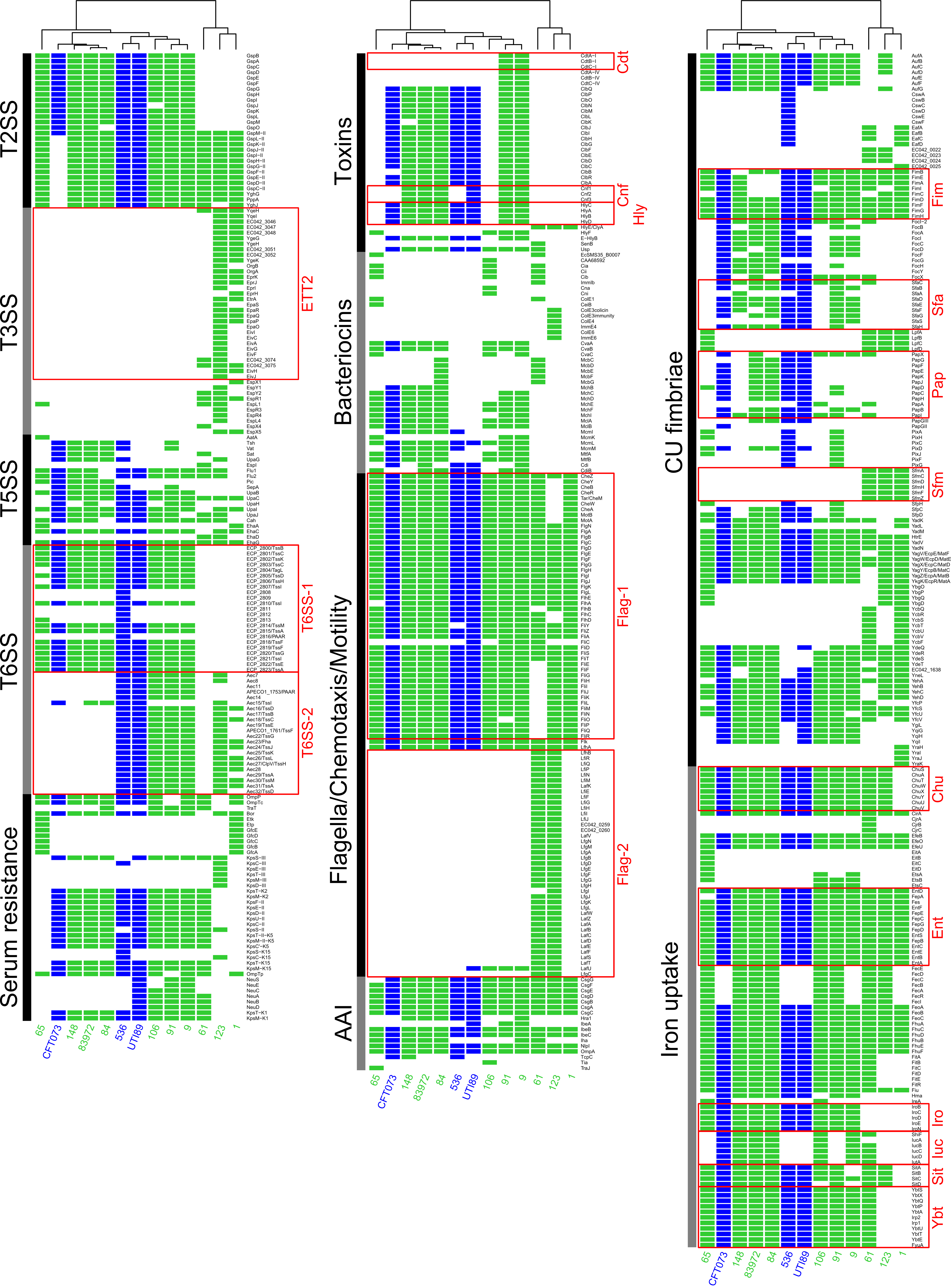

Supplement: FIGURE S3 — Heatmap indicating the presence or absence of ExPEC virulence- and fitness-associated factors including gene names/locus tags. Each row of the binary matrix indicates the presence or absence of a virulence/fitness-associated gene product (a BLASTP+ hit). Virulence/fitness factor classes are indicated at the side in black and grey. Strain names and individual columns are color-coded for ABU isolates (green) or UPEC strains (blue). Well-known ExPEC virulence determinants are indicated by red boxes. The clustering dendrogram attached to the heatmaps is based on the whole binary dataset of a best scoring ML tree with 1000 bootstrap resamplings. T2SS, type 2 secretion system; T3SS, type 3 secretion system; T5SS, type 5 secretion system; T6SS, type 6 secretion system; AAI, adhesion and invasion; CU fimbria, chaperone-usher fimbria. [file Image_3.TIF]

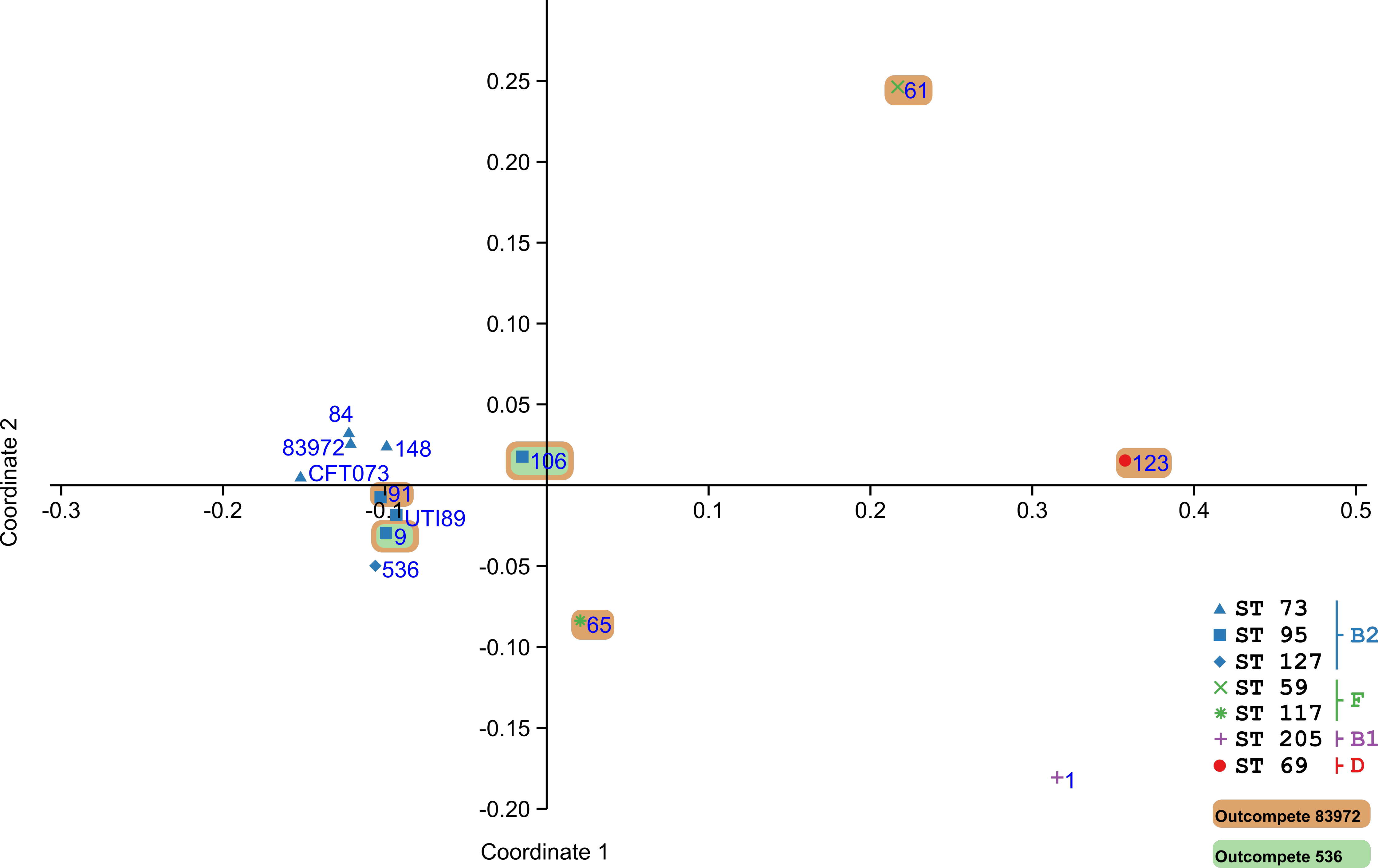

Supplement: FIGURE S4 — Principal Coordinates Analysis (PCoA) to examine the grouping of E. coli ABU isolates according to the presence/absence of fitness- and virulence-associated genes and their competitiveness in urine. The axes are scaled with eigenvalue scaling using the square root of the eigenvalue and indicate the percentage of variation explained in the PCoA. Strains of the same sequence type (ST) are indicated by the same symbol. Symbols are colored according to the phylogroup of each strain. Additionally, superior competitiveness of ABU isolates to model ABU strain 83972 and UPEC strain 563 is indicated. [file Image_4.TIF]
