# Supplementary material for: Characterization of Asymptomatic Bacteriuria Escherichia coli Isolates in Search of Alternative Strains for Efficient Bacterial Interference against Uropathogens
Source: Front Microbiol. 2018 Feb 14;9:214. doi: 10.3389/fmicb.2018.00214 (PMC5817090; doi:10.3389/fmicb.2018.00214)
Supplement: TABLE S1 — Bacterial strains used in this study. [file Table_1.DOCX]

**Table S1:** Bacterial strains used in this study

| Strain | Description | Reference |
| --- | --- | --- |
| *E. coli* ABU 1 | ABU isolate from a patient with diabetes | This study |
| *E. coli* ABU 9 | ABU isolate from a patient with diabetes | This study |
| *E. coli* ABU 61 | ABU isolate from a patient with diabetes | This study |
| *E. coli* ABU 65 | ABU isolate from a patient with diabetes | This study |
| *E. coli* ABU 84 | ABU isolate from a patient with diabetes | This study |
| *E. coli* ABU 91 | ABU isolate from a patient with diabetes | This study |
| *E. coli* ABU 106 | ABU isolate from a patient with diabetes | This study |
| *E. coli* ABU 123 | ABU isolate from a patient with diabetes | This study |
| *E. coli* ABU 148 | ABU isolate from a patient with diabetes | This study |
| *E. coli* 83972 | ABU model isolate, Orough:K5:H1 | ([Lindberg et al., 1975](#_ENREF_6)) |
| *E. coli* 83972::*cat* | Chloramphenicol-resistant variant of *E. coli* 83972 | ([Zdziarski et al., 2010](#_ENREF_12)) |
| *E. coli* MG1655 | Non-pathogenic laboratory strain, *E. coli* K-12 derivative | ([Blattner et al., 1997](#_ENREF_2)) |
| *E. coli* Nissle 1917 | Non-pathogenic, probiotic *E. coli* strain, O6:K5:H1 | ([Nissle, 1918](#_ENREF_9)) |
| *E. coli* CFT073 | UPEC model isolate, O6:K2:H1 | ([Mobley et al., 1990](#_ENREF_7)) |
| *E. coli* UTI89 | UPEC model isolate, O18:K1:H7 | ([Mulvey et al., 2001](#_ENREF_8)) |
| *E. coli* 536 | UPEC model isolate, O6:K15:H31 | ([Berger et al., 1982](#_ENREF_1)) |
| *E. coli* 536-21 | Non-hemolytic mutant of UPEC strain 536 | ([Hacker et al., 1983](#_ENREF_3)) |
| *E. coli* 119 | UPEC isolate | ([Kuch et al., 1982](#_ENREF_5)) |
| *E. coli* H1939 | FepA^+^, Fiu^-^, Cir^-^, FhuA^-^, FhuB^-^, *aroB*; enterobactin indicator *E. coli* strain | K. Hantke, University of Tübingen, Germany |
| *E. coli* H1887 | ColV^-^, Aer^-^, Iut^-^, FepA^-^, Fiu^-^, Cir^-^, *aroB*; aerobactin indicator *E. coli* strain | K. Hantke, University of Tübingen, Germany |
| *E. coli* K311 | Fecal isolate, pColV-K311, aerobactin-positive, enterobactin-positive | K. Hantke, University of Tübingen, Germany |
| *K. pneumoniae* 390 | Aerobactin-negative, enterobactin-positive | ([Trautmann et al., 1997](#_ENREF_11)) |
| *K. pneumoniae* W122 | Aerobactin-negative, enterobactin-negative | ([Podschun et al., 1993](#_ENREF_10)) |
| *E. coli* TB1 | *hsdR*-negative derivative of *E. coli* K-12 strain JM83 | New England Biolabs |
| *E. coli* TB1 / pC4003 | *E. coli* TB1 expressing the collagen-binding protein Pla of *Yersinia pestis* | ([Kienle et al., 1992](#_ENREF_4)) |

References:

Berger, H., Hacker, J., Juarez, A., Hughes, C., and Goebel, W. (1982). Cloning of the chromosomal determinants encoding hemolysin production and mannose-resistant hemagglutination in *Escherichia coli*. *J Bacteriol* 152(3)**,** 1241-1247.

Blattner, F.R., Plunkett, G., 3rd, Bloch, C.A., Perna, N.T., Burland, V., Riley, M., et al. (1997). The complete genome sequence of *Escherichia coli* K-12. *Science* 277(5331)**,** 1453-1462.

Hacker, J., Hughes, C., Hof, H., and Goebel, W. (1983). Cloned hemolysin genes from *Escherichia coli* that cause urinary tract infection determine different levels of toxicity in mice. *Infect Immun* 42(1)**,** 57-63.

Kienle, Z., Emődy, L., Svanborg, C., and O'Toole, P.W. (1992). Adhesive properties conferred by the plasminogen activator of *Yersinia pestis*. *J Gen Microbiol* 138 Pt 8**,** 1679-1687. doi: 10.1099/00221287-138-8-1679.

Kuch, B., Pál, T., and Emődy, L. (1982). Bacterial adherence and urinary tract infection. *The Lancet* 320(8289)**,** 107-108.

Lindberg, U., Hanson, L.A., Jodal, U., Lidin-Janson, G., Lincoln, K., and Olling, S. (1975). Asymptomatic bacteriuria in schoolgirls. II. Differences in *Escherichia coli* causing asymptomatic bacteriuria. *Act Paed Scand* 64(3)**,** 432-436.

Mobley, H.L., Green, D.M., Trifillis, A.L., Johnson, D.E., Chippendale, G.R., Lockatell, C.V., et al. (1990). Pyelonephritogenic *Escherichia coli* and killing of cultured human renal proximal tubular epithelial cells: role of hemolysin in some strains. *Infect Immun* 58(5)**,** 1281-1289.

Mulvey, M.A., Schilling, J.D., and Hultgren, S.J. (2001). Establishment of a persistent *Escherichia coli* reservoir during the acute phase of a bladder infection. *Infect Immun* 69(7)**,** 4572-4579. doi: 10.1128/IAI.69.7.4572-4579.2001.

Nissle, A. (1918). Die antagonistische Behandlung chronischer Darmstörungen mit Kolibakterien. *Med Klin* 2**,** 29-30.

Podschun, R., Sievers, D., Fischer, A., and Ullmann, U. (1993). Serotypes, hemagglutinins, siderophore synthesis, and serum resistance of *Klebsiella* isolates causing human urinary tract infections. *J Infect Dis* 168(6)**,** 1415-1421.

Trautmann, M., Ruhnke, M., Rukavina, T., Held, T.K., Cross, A.S., Marre, R., et al. (1997). O-antigen seroepidemiology of *Klebsiella* clinical isolates and implications for immunoprophylaxis of *Klebsiella* infections. *Clin Diagn Lab Immunol* 4(5)**,** 550-555.

Zdziarski, J., Brzuszkiewicz, E., Wullt, B., Liesegang, H., Biran, D., Voigt, B., et al. (2010). Host imprints on bacterial genomes - rapid, divergent evolution in individual patients. *PLoS Pathogens* 6(8)**,** e1001078. doi: 10.1371/journal.ppat.1001078.
