# Supplementary material for: Characterization of Asymptomatic Bacteriuria Escherichia coli Isolates in Search of Alternative Strains for Efficient Bacterial Interference against Uropathogens
Source: Front Microbiol. 2018 Feb 14;9:214. doi: 10.3389/fmicb.2018.00214 (PMC5817090; doi:10.3389/fmicb.2018.00214)
Supplement: TABLE S2 — Characteristics of the patients. [file Table_2.DOC]

**Table S2:** Characteristics of the patients

| ABU strain no. | Donor year of birth | Donor sex | Diabetes diagnosed | Diabetes type | Antibiotic treatment before isolation of the ABU isolate | Antibiotic treatment after isolation of the ABU isolate | No. of years with positive urine samples |
| --- | --- | --- | --- | --- | --- | --- | --- |
| 1 | 1959 | F | 2010 | 2 | No | No | 1 |
| 9 | 1990 | F | 2003 | 1 | No | Cefuroxime | 4 |
| 61 | 1992 | F | 2000 | 1 | No | Zinacef | 5 |
| 65 | 2000 | F | 2005 | 1 | No | Zinacef | 4 |
| 84 | 1991 | F | 1995 | 1 | No | Zinacef | 2 |
| 91 | 1987 | F | 1996 | 1 | No | No | 1 |
| 106 | 1992 | F | 2004 | 1 | No | Norfloxacin | 3 |
| 123 | 1998 | F | 2001 | 1 | No | Cefuroxime | 1 |
| 148 | 1989 | F | 1996 | 1 | No | No | 2 |

F, female
